# Bioactive Compounds from the Bornean Endemic Plant *Goniothalamus longistipetes*

**DOI:** 10.3390/antibiotics9120913

**Published:** 2020-12-16

**Authors:** Stephen P. Teo, Sanjib Bhakta, Paul Stapleton, Simon Gibbons

**Affiliations:** 1 Research Department of Pharmaceutical and Biological Chemistry, School of Pharmacy, University College London, 29-39 Brunswick Square, London WC1N 1AX, UK; p.stapleton@ucl.ac.uk (P.S.); s.gibbons@uea.ac.uk (S.G.); 2 Department of Biological Sciences, Institute of Structural and Molecular Biology, Birkbeck, University of London, Malet Street, London WC1E 7HX, UK; s.bhakta@bbk.ac.uk; 3 Forest Department Sarawak, Wisma Sumber Alam, Petra Jaya, Kuching 93600, Malaysia; 4 School of Pharmacy, University of East Anglia, Norwich Research Park, Norwich NR4 7TJ, UK

**Keywords:** *Goniothalamus longistipetes*, Annonaceae, antibacterial, efflux pumps, biofilm formation, bacterial conjugation, furo-pyrenone, styryllactones, alkaloid, alkenyl-hydroxybenzoic acid

## Abstract

The present study aimed to screen plants for bioactive compounds with potential antibacterial activities. In our efforts to evaluate plants from Borneo, we isolated and elucidated the structures of four natural products from the bioactive fraction of a chloroform extract of *Goniothalamus longistipetes* using various chromatographic and spectroscopic techniques. The bioactive compounds were identified as a known styryllactone, (+)-altholactone ((2*S*,3*R*,3a*S*,7a*S*)-3-hydroxy-2-phenyl-2,3,3a,7a-tetrahydrobenzo-5(4*H*)-5-one) (**1**), a new styryllactone, (2*S*,3*R*,3a*S*,7a*S*)-3-hydroxy-2-phenyl-2,3,3a,7a-tetrahydrobenzo-5(4*H*)-5-one) (**2**) as well as a new alkaloid, 2,6-dimethoxyisonicotinaldehyde (**3**) and a new alkenyl-5-hydroxyl-phenyl benzoic acid (**4**). **1** and **4** showed broad-spectrum anti-bacterial activities against Gram-positive and Gram-negative bacteria as well as acid-fast model selected for this study. Compound **2** only demonstrated activities against Gram-positive bacteria whilst **3** displayed selective inhibitory activities against Gram-positive bacterial strains. Additionally, their mechanisms of anti-bacterial action were also investigated. Using *Mycobacterium smegmatis* as a fast-growing model of tubercle bacilli, compounds **1**, **2** and **4** demonstrated inhibitory activities against whole-cell drug efflux and biofilm formation; two key intrinsic mechanisms of antibiotic resistance. Interestingly, the amphiphilic compound **4** exhibited inhibitory activity against the conjugation of plasmid pKM101 in *Escherichia coli* using a plate conjugation assay. Plasmid conjugation is a mechanism by which Gram-positive and Gram-negative-bacteria acquire drug resistance and virulence. These results indicated that bioactive compounds isolated from *Goniothalamus longistipetes* can be potential candidates as ‘hits’ for further optimisation.

## 1. Introduction

The search for and use of drugs derived from natural sources has furnished mankind with a rich and important source of therapeutic agents. For instance, Newman et al. [1] reported that from 1981 to 2002, 75% of all the anti-infective drugs approved for use were derived from natural sources. Additionally, 61% of all new chemical compounds introduced globally as drugs during the same period were derived from, or were inspired by natural products [2]. Plants have been a source of antibiotics in both ancient and modern times [3].

With the emergence of multi-drug-resistance in WHO-priority bacterial pathogens, the search for new and more effective cures is imperative [4]. Resistance to antimicrobials is a growing and significant problem and affects treatment options. As many existing antibiotics or their synthesized derivatives become ineffective as a result of resistance, antimicrobials and other compounds derived from plants can offer an alternative and novel source [5].

The tropical rain forests of Borneo are one of the global hotspots for plant biodiversity, many of which have traditional uses. Although there is no specific information on ethnobotanical uses for *Goniothalamus longistipetes* which can be attributed to the rareness of this endemic species, other more widespread species of the genus *Goniothalamus* from the Annonaceae family are known to possess ethnobotanical uses in Borneo including as folkmedicines [6,7]. Wiart [8] and Muhammad et al., [9] have reviewed *Goniothalamus* phytochemistry for antibacterial properties and both noted that the genus was poorly studied but with potential for antibacterial compounds whilst the main phytochemicals isolated were styryllactones [8,9] and acetogenins [8,10]. In this study, we screened the stem bark of *Goniothalamus longistipetes* for these activities and report the structures and potential antibacterial action of the compounds.

## 2. Results

### 2.1. Phytochemistry

**Figure 1:**
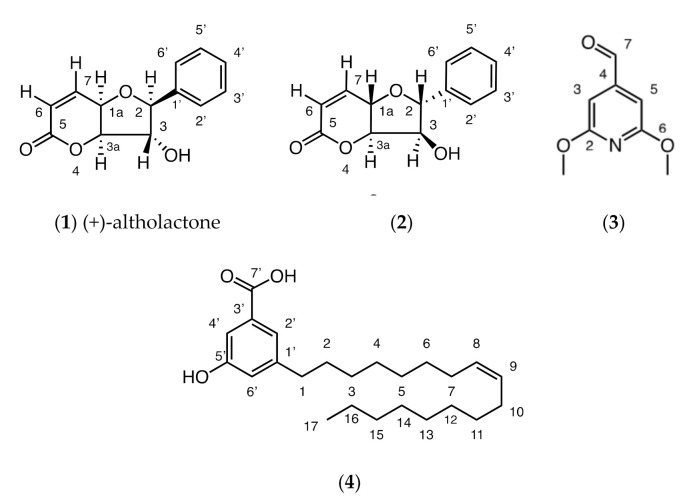
Compounds isolated from *Goniothalamus longistipetes*—a known styryllactone, (+)-altholactone ((2*S*,3*R*,3a*S*,7a*S*)-3-hydroxy-2-phenyl-2,3,3a,7a-tetrahydrobenzo-5(4*H*)-5-one) (**1**), a new styryllactone, (2*S*,3*R*,3a*S*,7a*S*)-3-hydroxy-2-phenyl-2,3,3a,7a-tetrahydrobenzo-5(4*H*)-5-one) (**2**) as well as a new alkaloid, 2,6-dimethoxyisonicotinaldehyde (**3**) and a new alkenyl-5-hydroxyl-phenyl benzoic acid (**4**).

The bioactive fraction of the chloroform extract of *Goniothalamus longistipetes* gave a known (**1**) and new styryllactone (**2**) as well as a new pyridine alkaloid (**3**) and a new alkenyl-5-hydroxy-phenyl benzoic acid (**4**) (Figure 1).

A full structure elucidation of these compounds is presented in the Supplementary Materials (Figures S1–S33; Tables S1–S4). Compound **1** was identified by comparison of its ^1^H, ^13^C and mass spectral data with that of the literature [11]. The structures of **2**–**4** were determined by full 2D NMR analysis, particularly using HMBC spectroscopy. In addition to these techniques, for compound **4**, the position of the double bond was identified by derivatization and comparison of fragments with a mass spectral library, confidently allowing placement of the double bond between C-8 and C-9.

### 2.2. Antibacterial Activities

Compounds **1** and **4** showed broad-spectrum anti-bacterial activities against Gram-positive, Gram-negative as well as acid-fast mycobacterial strains selected for this study, while **2** demonstrated activities only against Gram-positives and a strain of *Mycobacterium* sp. **3** exhibited specific inhibitory activities against Gram-positive bacterial strains. The anti-bacterial activities of all the compounds and their respective selective indices are listed in Table 1. For the broth dilution assay for Gram-positive and negative-bacteria, inhibitors were serially (2-fold) diluted with a starting concentration of 512 µg/mL whilst for the acid-fast mycobacterial strains, a serial dilution ranging from 500 µg/mL to 0.0224 µg/mL was carried out.

Compound **1** exhibited more toxicity to the eukaryotic cell-line (RAW 264.7) compared to bacterial cells (Table 1: selectivity indices/S.I. < 1) except for *M. bovis* BCG, a slow-growing model of *M. tuberculosis*. We can infer from this result that compound **1** is selective (Table 1: selectivity indices/S.I. = 4) in inhibiting slow-growing mycobacterial growth. Compound **2** was found to be selective for inhibiting growth of all strains of *Staphylococcus* sp. (Table 1: selectivity indices/S.I. = 3.91) used in this study. Whereas compound **2** was found to be equally toxic to the eukaryotic cell when it was tested against the Gram-negative and mycobacterial species. Interestingly, compound **3** was selectively potent (Table 1: selectivity indices/S.I. = 3.91) against the *Bacillus subtilis* strain. Compound **4** displayed the best selective inhibitory growth against the Gram-positive and acid-fast strains (Table 1: selectivity indices/S.I. = 16–32). In this whole-cell drug susceptibility assay, the mouse macrophage cell line (RAW 264.7) was used for the evaluation of toxicity against eukaryotic cells.

### 2.3. Drug Accumulation and Inhibition of Biofilm Formation Assays

**Figure 2:**
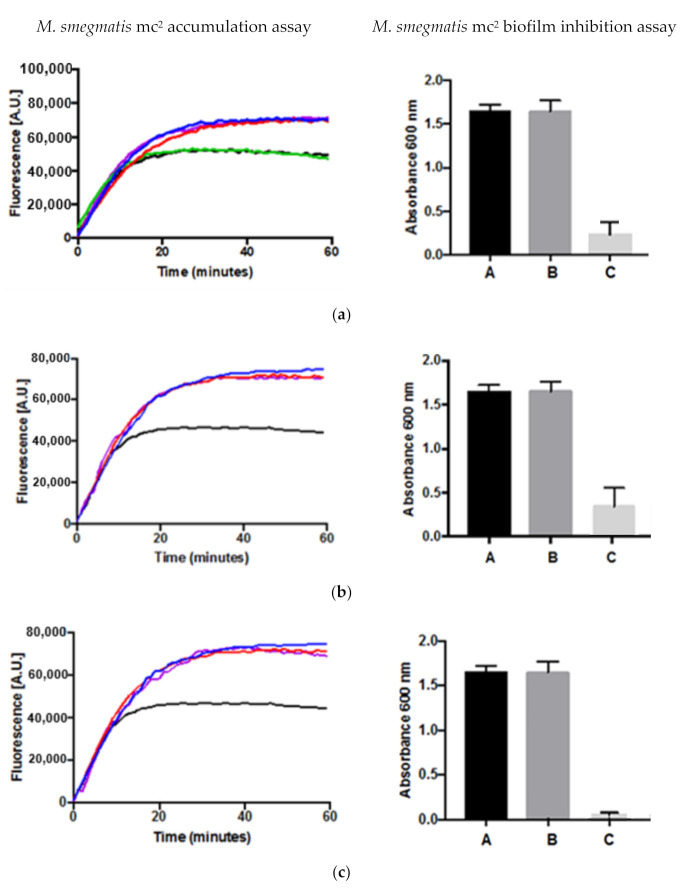
Inhibitory activities by compound (**a**) **1**, (**b**) **2** and (**c**) **4** in both the accumulation (left) using 3 replicates (coefficient of variation, cv < 1) and biofilm formation (right ) using 6 replicates (*p*-value < 0.01) assays. Blue line: verapamil (0.5 × MIC)—A—negative control; Red line: chlorpromazine 0.5 × MIC—B—DMSO; Green line: compound with no known activity (0.5 × MIC)—C—0.5 × MIC; Purple line: compound tested for activity (0.5 × MIC); Black line: drug-free control.

Efflux pumps and biofilm formation are two important mechanisms by which bacteria develop resistance to antibiotics. The results of the accumulation assay and the biofilm formation assay for compounds **1**, **2** and **4** are shown in Figure 2. Compounds **1**, **2** and **4** displayed inhibitory activities in the whole cell phenotypic accumulation assay using *M*. *smegmatis* as a model when compared to the controls demonstrating that the compounds can inhibit the efflux pumps. For the biofilm inhibition assay, the three compounds also exhibited inhibitory activities (*p*-value < 0.01) at both the concentrations for MIC and their respective sub-inhibitory concentrations (half that of MICs) when compared with their respective controls. For the accumulation assay, EtBr was used as a known substrate for whole cell efflux pumps and the two known efflux pump inhibitors, verapamil and chlorpromazine, were used as controls whilst for the biofilm inhibition assay, a negative control was employed. DMSO was also used to see if there was any effect on biofilm inhibition, because DMSO was used as the solvent to dissolve the compounds tested.

### 2.4. Plate Conjugation Assay

Apart from efflux pumps and biofilm formation, another mechanism that contributes to drug resistance in bacteria is plasmid conjugation. Bacteria can disseminate plasmids harbouring genes responsible for drug resistance to other bacteria through plasmid conjugation [12]. Inhibitor of bacterial conjugation for specific plasmids such as pKM101 can therefore be of use to reduce drug resistance. Amphiphilic compounds are known to inhibit bacterial plasmid conjugation and as compound **4** is amphiphilic, a plate conjugation assay [12] was carried out in order to determine its inhibitory effect on bacterial plasmid conjugation in *Escherichia coli*.

**Figure 3:**
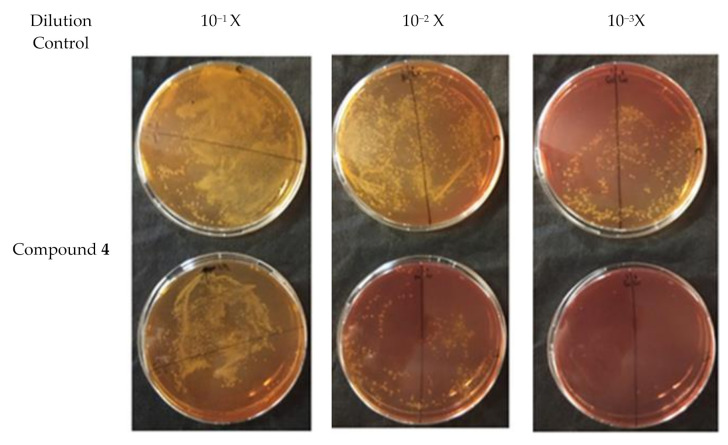
Result of the plate conjugation assay showing the transconjugant cells for **4** with a concentration of a quarter of MIC and control using plasmid pKM101 in *E. coli*.

**Figure 4:**
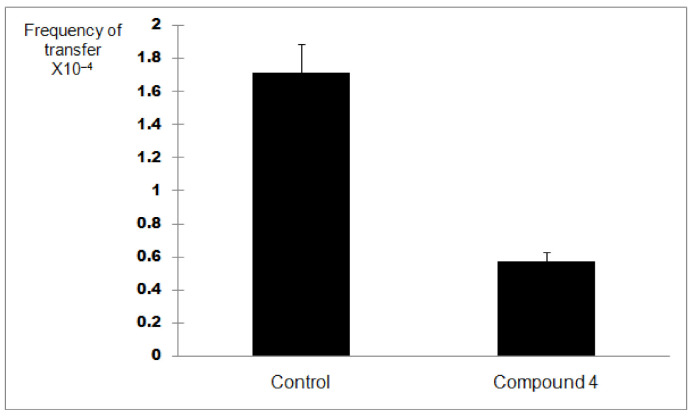
Bar graph showing the inhibitory activity on pKM101 plasmid conjugation of compound **4** in *E. coli* when compared with the control (*p* < 0.05). The transfer frequency is expressed as the number of transconjugants per donor cell obtained from mating.

In order to discern between antibacterial activity and the inhibition of plasmid conjugation, compound **4** was assessed at a quarter of its MIC value (256 µg/mL) for this assay. A negative control was employed. Plasmid pKM101 (donor strain) confers resistance to amoxicillin whilst the recipient strain ER1793 is resistant to streptomycin. Subculturing onto media containing amoxicillin and streptomycin allows determination of transconjugation. So, each strain was subcultured in its selective media based on resistance characteristics. The experiment was repeated three times. The results of the conjugation assay are shown in Figure 3. Based on the result of the transconjugant bacterial colony count as well as the donor colony count in the control, there was an approximately 70% reduction in plasmid conjugation when compared to the control (*p* < 0.05) (Figure 4) indicating that compound **4** might be able to inhibit plasmid conjugation.

## 3. Discussion

There are few studies done on the antibacterial properties for chemical constituents isolated from species of *Goniothalamus*. Styryllactone, altholactone, and previously isolated from *G. malayanus* showed moderate broad-spectrum activities against Gram-positive and Gram-negative bacteria except *Pseudomonas aeruginosa* [13] which is consistent with the results from our study. Another stereoisomer of compound **1**, isoaltholacthone, isolated from *Goniothalamus grandifolius* (not isolated from this study) also displayed broad-spectrum activities [14]. For the pyrimidine alkaloid (**3**) isolated in this study and for the first time from *Goniothalamus,* specific activities against strains of Gram-positive bacteria were only observed. Previously, only aristolactam alkaloids have been isolated from *Goniothalamus velutinus*, which did not show any antibacterial activities [15]. Studies on the mechanisms of action of antibacterial compounds from *Goniothalamus* have been neglected.

The results of the inhibitory activities in the accumulation assay for compounds **1**, **2** and **4** (Figure 3) indicated that they were able to inhibit efflux pumps. In addition, all three compounds could also inhibit the formation of biofilms using *M. smegmatis* as a model and there is a correlation with efflux pump inhibition. There is evidence from several studies that have shown that efflux pumps also play a significant role in biofilm formation [16]. Kvist et al. [17] demonstrated in *E. coli* that the inactivation of efflux pumps resulted in the failure to form a bacterial biofilm. The downregulation in particular of, the *AdeFGH* efflux pump, is probably responsible for a decrease in the formation of biofilm in *Acinetobacter baumannii* treated with tigecycline below inhibitory concentrations [18]. Aristolactam alkaloids from *Goniothalamus velutinus* have been shown to possess selective inhibitory activities on biofilm formation of some selected Gram-positive and Gram-negative bacterial species [17].

Because there is a correlation between efflux pumps and inhibition of biofilm, inhibitors of efflux pumps could potentially act as inhibitor of biofilm formation. By understanding the roles that efflux pumps play in biofilm formation, it is possible that novel therapeutic strategies can be developed to inhibit and interfere with biofilms and therefore improve the treatment of bacterial infections [18].

Stereoisomers **1** and **2** showed differing activities against Gram-positive, Gram-negative and acid-fast mycobacterial strains. However, both stereoisomers did not show much difference in their activities against the two effluxing strains (XU212 and SA1199B) for *S. aureus* when compared with the susceptible strain (SA25923), indicating that they might not be able to inhibit the efflux pumps in both strains. The overexpression of efflux pumps in XU212 is coded by the *tet*(K) gene [19] and causes resistance to the antibiotic tetracycline while in SA1199B, it is coded by the *norA* gene which causes overexpression of the NorA efflux pump and therefore resistance to the antibiotic norfloxacin and both efflux pumps belong to the Major Facilitator Superfamily (MFS) family [20]. However, in the whole cell phenotypic accumulation assay using *Mycobacterium smegmatis*, there were good activities shown by both styryllactone isomers. This could mean that the compounds inhibit efflux pumps other than those in the MFS family, because *Mycobacterium smegmatis* is known to possess all 5 families of efflux pumps.

The pKM101 plasmid belongs to the IncN plasmid family, and is a 35.4kb derivative from the parent R46 [21]. It renders *E. coli* and *S. typhimurium* resistant to the effects of UV radiation and mutagenesis [21]. The DNA region of R46, when removed *in vivo*, removed the gene coding for resistance to sulphonamides, streptomycin, and spectinomycin but conferred the ability of pKM 101 to increase resistance to UV as well as the presence of ampicillin resistance and the fertility factor of *E. coli* [22].

The result from the plate conjugation assay for compound **4** showed a reduction in the plasmid conjugation by about 70%. This demonstrated that the compound which is amphiphilic might be able to inhibit the conjugation process (e.g.,Type-IV secretory systems (T4SS)) or the horizontal transfer of the plasmid [21] that carried the resistance gene (pKM101) which is resistance to amoxicillin. The mechanism behind the inhibition of plasmid conjugation by **4** was not investigated. However, compounds very similar to compound 4 (5-alkyl resorcinols) isolated from the Australian plant, *Hakea amplexicaulis,* were found to be able to cleave DNA in the presence of Cu^2+^ [22]. Type-IV secretion systems (T4SS) are important secretion systems or ‘molecular syringes’ that are found in both Gram-positive and Gram-negative bacteria and secrete a wide range of substrates, from single proteins to protein–protein and protein–DNA complexes into other cells in order to manipulate and often kill rival bacterial and eukaryotic cells. [21,23]. The horizontal gene transfer conferred by T4SS is also one of the main mechanisms for the spread of genes responsible for resistance to antibiotics as well as virulence factors in a variety of human diseases such as whooping cough (*Bordetella pertussis*) and peptic and gastric cancers [24]. Plasmids carry resistance genes which are separated from and can replicate independently of the bacterial chromosomal DNA and can, therefore, render resistance to one or more antibiotics [24,25,26]. Plasmids are commonly able to transfer between bacterial cells from different species by a mechanism known as conjugation, which involves cell-to-cell contact followed by transfer of a copy of plasmid DNA from a donor to a recipient [23]. The spread of multiple antimicrobial resistance has been enhanced by selective pressure from human and veterinary medicine [27].

## 4. Materials and Methods

### 4.1. General Experimental Procedures

One- and two-dimensional (1D and 2D) NMR spectra were recorded on a 500 MHz spectrometer (Bruker) using tetramethylsilane as internal references with referencing to solvent signals (CDCl_3_). UV and IR spectra were recorded on a Thermo Electron Corporation Helios spectrophotometer and on a Nicolet 360 FT-IR spectrophotometer respectively. Mass spectra were recorded using HRESIMS for accurate mass determination (m/z value up to four decimal places) and were performed using a Waters Quadrupole/Time of flight (Q-ToF) premier Tandem Mass Spectrometer and a Shimadzu LCMS-2020 which is a single quadrupole mass spectrometer with a mass range of 50 Da to 2000 Da. Chemical and biological reagents were obtained from Sigma Aldrich Company Ltd., Dorset, UK unless otherwise stated.

### 4.2. Plant Materials

The bark of *G. longistipetes* (approximately 0.5 kg) was obtained in Sarawak, Malaysia in February 2013. The plant specimen was identified at the Forest Herbarium, Forest Research Centre, Forest Department Sarawak, Malaysia and a voucher specimen (S.90) was kept at the Forest Herbarium as well as the herbarium at the School of Pharmacy, University College London.

### 4.3. Extraction and Isolation

The pulverized bark of *G. longistipetes* was extracted in the sequential order—hexane and chloroform and methanol using the Soxhlet apparatus. The respective crude extracts were concentrated and dried by means of a rotatory evaporator and blow down under nitrogen gas and then fractionated by Vacuum Liquid Chromatography (silica gel with 100–200 mesh as stationary phase) into 13 fractions using a mixture of two solvents in increasing order of polarity. Preparative-TLC (prep-TLC) was done on the fractions based on the activities in the biological assay using a full normal-phase silica sheet gel 60 F_254_ (Merck KGaA).The TLC plate was then air dried in a flow hood and viewed under short UV (254 nm) and long UV (356 nm) light (Camag) as well as sprayed with appropriate stains to locate the compounds. The respective bands were then scratched off the plate and the compounds were obtained by desorption using a solvent appropriate for the pure compounds.

### 4.4. Bacterial Strains and Drug Susceptibility Assays

Broth dilution assay [28] was used for Gram-positive and Gram-negative bacteria while spot culture growth inhibition assay (SPOTi) [29,30] was employed for acid-fast strains. The Gram-positive bacterial strains used were *Staphylococcus aureus:* SA25923 (susceptible strain), XU212 (tetracycline-resistant), SA1199B (norfloxacin-resistant), eMRSA-15 (methicillin-resistant) and one *Bacillus* species: *Bacillus subtilis* (a susceptible strain) whilst 3 susceptible strains of Gram-negative bacteria were *E. coli* NCTC 10418, *Pseudomonas aeruginosa* and *Klebsiella pneumoniae* NCTC 10662.

For mycobacteria, *Mycobacterium smegmatis* mc^2^155 (fast growing) and *Mycobacterium bovis* BCG Pasteur ATCC35734 (slow growing) species were used in this study. A serial dilution in a 96-well microtiter plate was done for the compounds in DMSO.

### 4.5. Eukaryotic Cytotoxicity Assay

Mammalian cells (mouse macrophage RAW 264.7 (ATCC TIB71)) used in the cytotoxicity assay were a kind gift from Professor Simon Gordon, University of Oxford. The rezazurin assay method was employed for the cytotoxicity assay [31]. The 90% growth inhibitory concentration (GIC_90_) was ascertained and the selectivity index (SI), was determined as the ratio between the GIC_90_ on RAW 264.7 mouse macrophages and the MIC on the respective bacterial strain. For example, the SI = GIC_90_/MIC. Paclitaxel was used as the control for the cytotoxic assay.

### 4.6. Accumulation Assay

The accumulation assay was conducted using the whole cell phenotypic assay with *Mycobacterium smegmatis* mc^2^ 122 cells as the model. The method used followed that employed by [32]. Verapamil (final working concentration of 12.5 µg/mL) and chlorpromazine (final working concentration of 15.6 µg/mL) were the positive efflux pump inhibitory controls together with a drug-free control in the assay. All the test samples and blanks were prepared in triplicate. The continuous live monitoring of the florescence was done, a cycle of measurement every 60 s for a total period of 60 min, using a plate reader (FLUOstar OPTIMA, BMG Labtech, Manchester, UK) with the settings as follows; 540 and 590 nm for excitation and detection of fluorescence (emission).

### 4.7. Biofilm Assay

The biofilm assay followed the method applied by [33] by means of the whole cell phenotypic assay using *M. smegmatis* mc^2^ 122 as a model. Compounds at the concentrations of MICs and sub-inhibitory concentration (half of MICs) were tested. In addition, experiments using DMSO at the concentration used to dissolve the tested compounds were also used to determine if there was any effect on biofilm formation. Additionally, a negative experiment was also set up.

### 4.8. Plate Conjugation Assay

The plate-conjugation assay used the broth mating method as employed by [12]. Two bacterial strains of *E. coli* were used in this assay for the plasmid conjugation assay, as shown in Table 2.

The ER1793 strain was used as the recipient (streptomycin-resistant) whilst the pKM101-containing strain was the donor (amoxicillin-resistant). The plate conjugation assay used MacConkey agar supplemented with amoxicillin to facilitate identification of donor cells, and agar containing amoxicillin and streptomycin to distinguish transconjuants. The number of transconjugants to donor cells were used to determine the conjugation frequencies. Conjugation efficacy was calculated based on the conjugation frequencies expressed as a percentage relative to the control.

### 4.9. Statistical Analyses

All statistical analyses were computed using Microsoft Excel 2010 or GraphPad prism. The coefficient of variation (cv) was calculated as a ratio of the standard deviation to the mean with the data obtained from the 3 replicates (no outlier if cv < 1) whilst the *p*-value was carried out by comparing the means of each experiment using independent t-test (significant difference if *p*-value < 0.05).

## 5. Conclusions

This study highlighted the potential of bioactive compounds from *Goniothalamus longistipetes* with reasonably good activities as drug ‘hits’. Compound **1** as well as **2** and **3** can be further optimized especially against the slow-growing mycobacterium and Gram-positive bacterial strains respectively whilst the broad-spectrum compound **4** deserves further investigation and development for its inhibitory activities on the 3 key resistance determinants.

## Figures and Tables

**Table 1:** Antibacterial (MIC) and cytotoxic (IC_50_) activities of compounds **1**–**4** and their respective selectivity indices (S.I.).

	1	S.I.	2	S.I.	3	S.I.	4	S.I.
**Gram-positive bacteria**								
*Staphylococcus aureus*								
SA25923	64.0 µg/mL (275.7 µM)	0.49	128 µg/mL (551.6 µM)	3.91	128 µg/mL (703.1 µM)	1.95	8 µg/mL (21.4 µM)	32
SA1199B	64.0 µg/mL (275.7 µM)	0.49	128 µg/mL (551.6 µM)	3.91	128 µg/mL (703.1 µM)	1.95	8-16 µg/mL (21.4–42.7 µM)	16
XU212	64.0 µg/mL (275.7 µM)	0.49	128 µg/mL (551.6 µM)	3.91	128 µg/mL (703.1 µM)	1.95	8-16 µg/mL (21.4–42.7 µM)	16
eMRSA-15	64.0 µg/mL (275.7 µM)	0.49	128 µg/mL (551.6 µM)	3.91	128 µg/mL (703.1 µM)	1.95	8-16 µg/mL (21.4–42.7 µM)	16
*Bacillus subtilis*	32.0 µg/mL (137.9 µM)	0.98	256 µg/mL (551.6 µM)	1.95	64 µg/mL (363.5 µM)	3.91	8-16 µg/mL (21.4–42.7 µM)	16
**Gram-negative bacteria**								
*E. coli*	256 (1103.0 µM)	0.12	512 (2206.0 µM)	0.98	256µg/mL (1406.2 µM)	0.98	512 µg/mL (1367.9 µM)	0.5
*Klebsiellapnuemoniae*	256 (1103.0 µM)	0.12	512 (2206.0 µM)	0.98	256 µg/mL (1406.2 µM)	0.98	128 µg/mL (342.0 µM)	2
*Pseudomonas aeruginosa*	500µg/mL (2206.0 µM)	0.6	512µg/mL (2206.0 µM)	0.98	256µg/mL (1406.2 µM)	0.98	128 µg/mL (342.0 µM)	2
**Acid-fast mycobacteria**								
*Mycobacterium smegmatis*	>500 µg/mL (2154.5 µM)	<0.06	500 µg/mL (2154.5 µM)	1	500 µg/mL (2154.5 µM)	0.5	n.a.	n.a.
*Mycobacterium bovis* BCG	7.81 µg/mL (33.6 µM)	4.0	500 µg/mL (2154.5 µM)	1	500 µg/mL (2154.5 µM)	0.5	7.8 µg/mL (20.9 µM)	32
**Cell line**								
RAW 264.7 macrophage cells	31.25 µg/mL		500 µg/mL		250 µg/mL		250 µg/mL	

n.a.—not available

**Table 2:** The donor and recipient strains used in the conjugation study.

Donor Strain	Plasmid	Resistance Marker	Recipient Strain	Resistance Marker
WP2	pKM101	Amoxicillin	ER1793	Streptomycin

## Supplementary Materials

The following are available online at https://www.mdpi.com/2079-6382/9/12/913/s1, Figure S1: ^1^H NMR spectrum of **1** in CDCl_3_ (500 MHz); Figure S2: ^1^H NMR spectrum of **1** (expanded) in CDCl_3_ (500 MHz); Figure S3: ^1^H NMR spectrum of **1** (expanded) in CDCl_3_ (500 MHz); Figure S4: ^13^C NMR spectrum of **1** in CDCl_3_ (500 MHz); Figure S5: Expansion of HMQC spectrum of **1** in CDCl_3_; Figure S6: Expansion of HMBC spectrum of **1** in CDCl_3_; Figure S7: Expansion of COSY spectrum of **1** in CDCl_3_; Figure S8: ESI-MS spectrum of **1** showing peak ion at m/z 238.1 [M-H]^-^ (indicated by arrow); Figure S9: ^1^H NMR spectrum of **2** in CDCl_3_ (500 MHz); Figure S10: ^1^H NMR spectrum of **2** in CDCl_3_ (500 MHz); Figure S11: ^1^H NMR spectrum of **2** in CDCl_3_ (500 MHz); Figure S12: ^13^CNMR spectrum of **2** in CDCl_3_ (125 MHz); Figure S13: HMQC spectrum of **2** in CDCl_3_; Figure S14: HMBC spectrum of **2** in CDCl_3_; Figure S15: COSY spectrum of **2** in CDCl_3_; Figure S16: HRMS spectrum of **2** showing peak ion at m/z 255.0627; Figure S17: ^1^H NMR spectrum of **3** in CDCl_3_ (500 MHz); Figure S18: ^13^C spectrum of **3** in CDCl_3_ (125 MHz); Figure S19: ^13^C spectrum of **3** in CDCl_3_ (125 MHz); Figure S20: HMQC spectrum of **3** in CDCl_3_; Figure S21: HMBC spectrum of **3** in CDCl_3_; Figure S22: COSY spectrum of **3** in CDCl_3_; Figure S23: ESI-MS spectrum of **3** showing peak ion at m/z 166.0 (indicated by arrow); Figure S24: ^1^H NMR spectrum of **4** in CDCl_3_ (500 MHz); Figure S25: ^13^C NMR spectrum of **4** in CDCl_3_ (125 MHz); Figure S26: ^13^C NMR spectrum of **4** (expanded) in CDCl_3_ (125 MHz); Figure S27: HMQC spectrum of **4** in CDCl_3_; Figure S28: HMBC spectrum of **4** in CDCl_3_; Figure S29: COSY spectrum of **4** in CDCl_3_; Figure S30: HRMS spectrum of **4** showing peak ion at m/z 373.2732; Figure S31: The EI mass spectrum of the GC peak corresponding to the alkenyl chain of 4 as identified from the NIST database (fragmentation pattern proposed as oleic acid trimethylsilyl acid); Figure S32: Preparation of dimethyl disulphide derivatives; Figure S33: Derivatisation of the hydroxyl groups; Table S1: ^1^H (500 MHz) and ^13^C NMR (125 MHz) and HMBC spectroscopic data of **1** recorded in CDCl_3_ compared with published data; Table S2: Comparison of the hydrogen chemical shifts and coupling constants between **2** with 3 other known styryllactones; Table S3: ^1^H (500 MHz) and ^13^C NMR (125 MHz) and HMBC spectroscopic data of 2 recorded in CDCl3; Table S4:. ^1^H (500 MHz),^13^C NMR (125 MHz) and HMBC spectroscopic data of **3** and **4** recorded in CDCl3.
