# Supplementary material for: Bioactive Compounds from the Bornean Endemic Plant Goniothalamus longistipetes
Source: Antibiotics (Basel). 2020 Dec 16;9(12):913. doi: 10.3390/antibiotics9120913 (PMC7767169; doi:10.3390/antibiotics9120913)
Supplement: Supplementary file 1 [file antibiotics-09-00913-s001.pdf]

antibiotics

MDPI

Supplementary Materials

Article

Bioactive Compounds from the Bornean Endemic Plant *Goniothalamus longistipetes*

Stephen P. Teo [1,2,3,*], Sanjib Bhakta [2], Paul Stapleton [1] and Simon Gibbons [1,4]

[1] Research Department of Pharmaceutical and Biological Chemistry, School of Pharmacy, University College London, 29-39 Brunswick Square, London WC1N 1AX, UK; p.stapleton@ucl.ac.uk (P.S.); s.gibbons@uea.ac.uk (S.G.)

[2] Department of Biological Sciences, Institute of Structural and Molecular Biology, Birkbeck, University of London, Malet Street, London WC1E 7HX, UK; s.bhakta@bbk.ac.uk

[3] Forest Department Sarawak, Wisma Sumber Alam, Petra Jaya, Kuching 93600, Malaysia

[4] School of Pharmacy, University of East Anglia, Norwich Research Park, Norwich NR4 7TJ, UK

* Correspondence: stephetp@sarawak.gov.my

Received: 28 September 2020; Accepted: 15 December 2020; Published: date

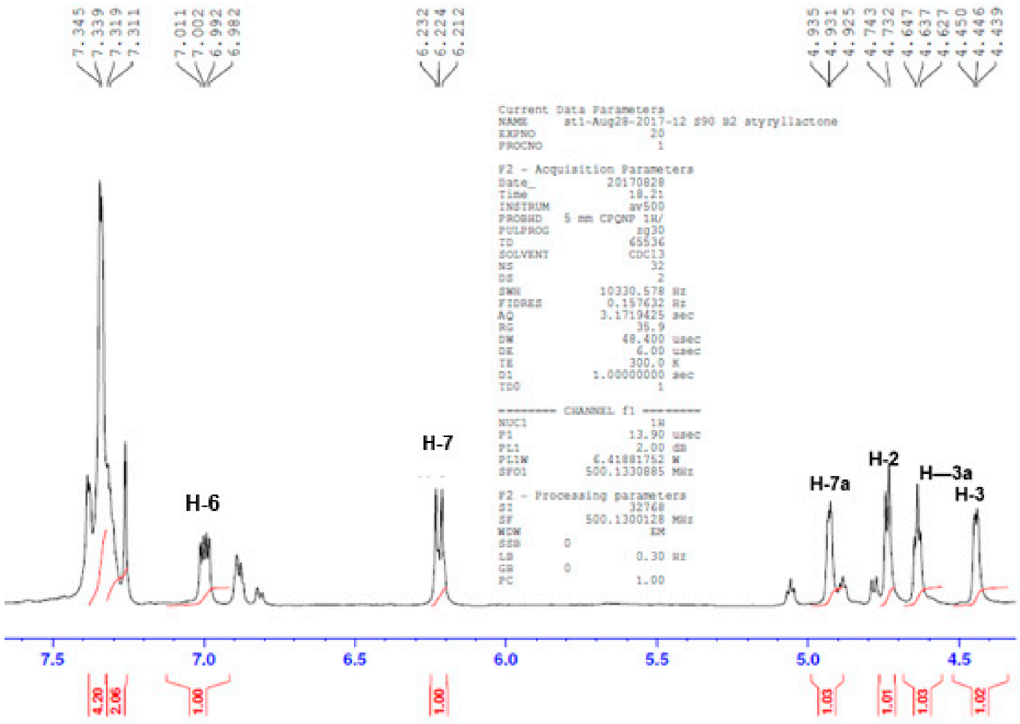

Figure S1 ^{1}H NMR spectrum of **1** in $CDCl_3$ (500 MHz)

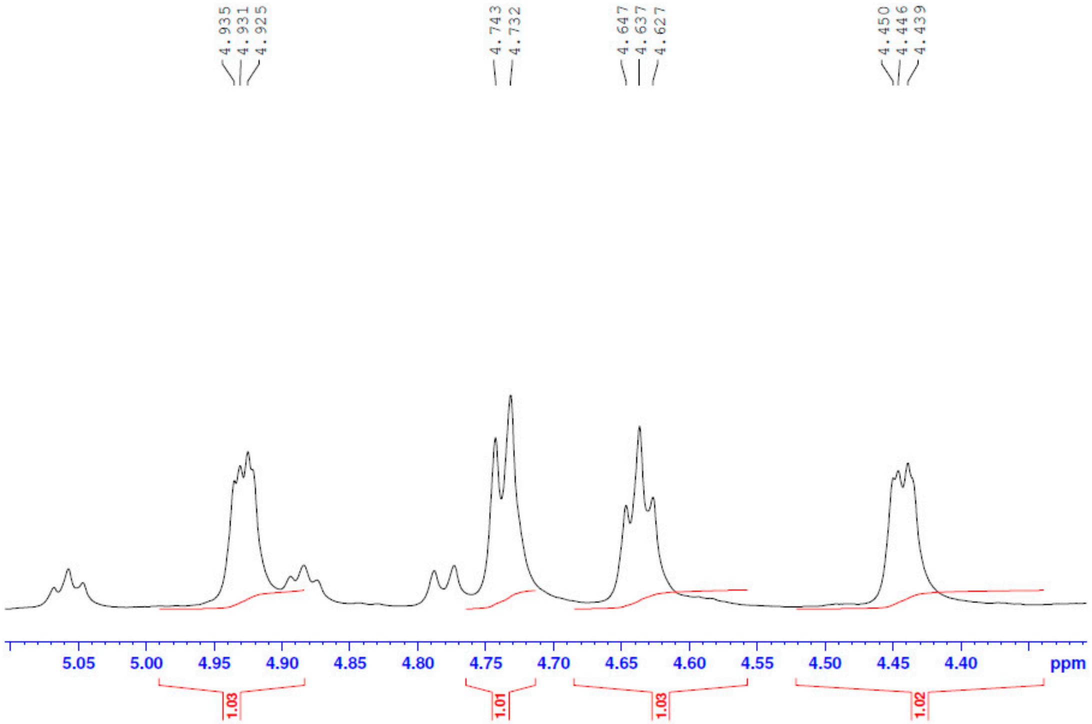

Figure S2 ^{1}H NMR spectrum of **1** (expanded) in $CDCl_3$ (500 MHz)

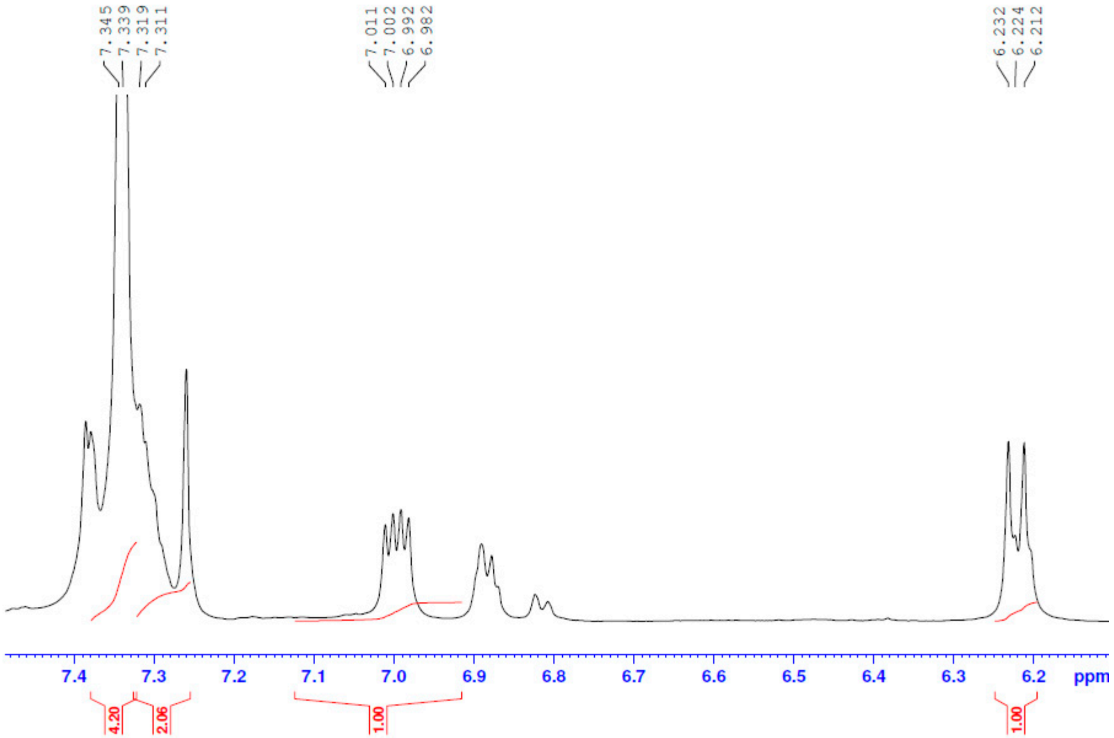

Figure S3 ^{1}H NMR spectrum of **1** (expanded) in $CDCl_3$ (500 MHz)

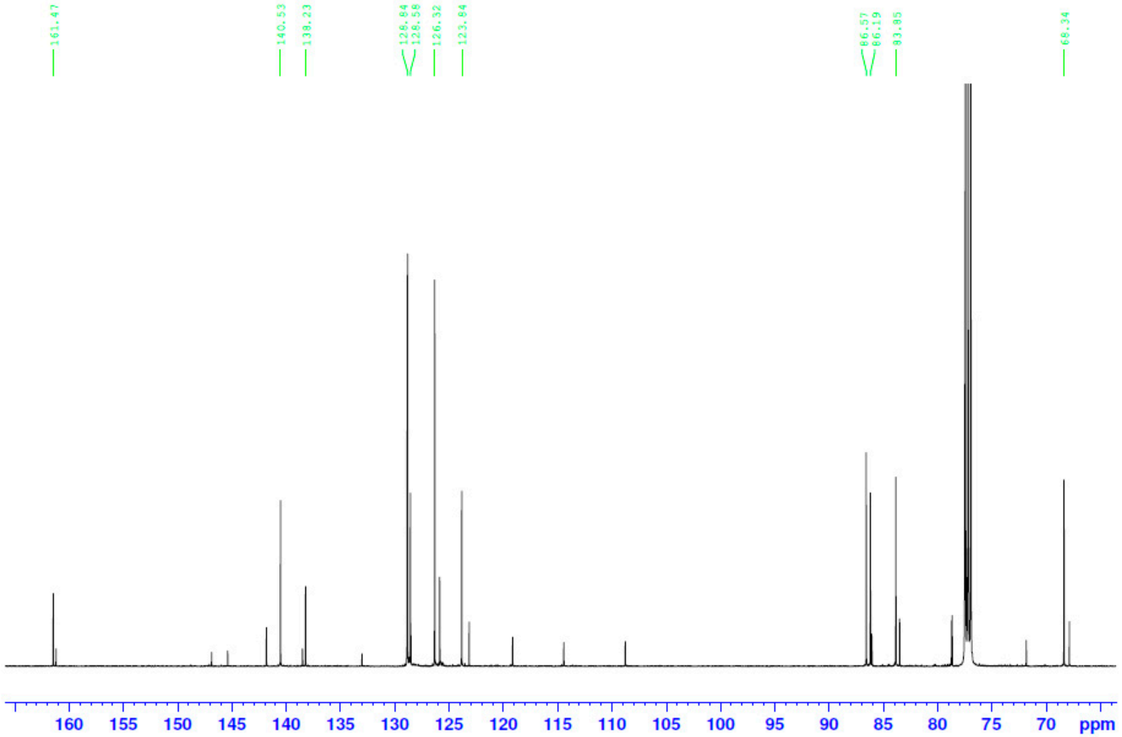

Figure S4 ^{13}C NMR spectrum of **1** in $CDCl_3$ (500 MHz)

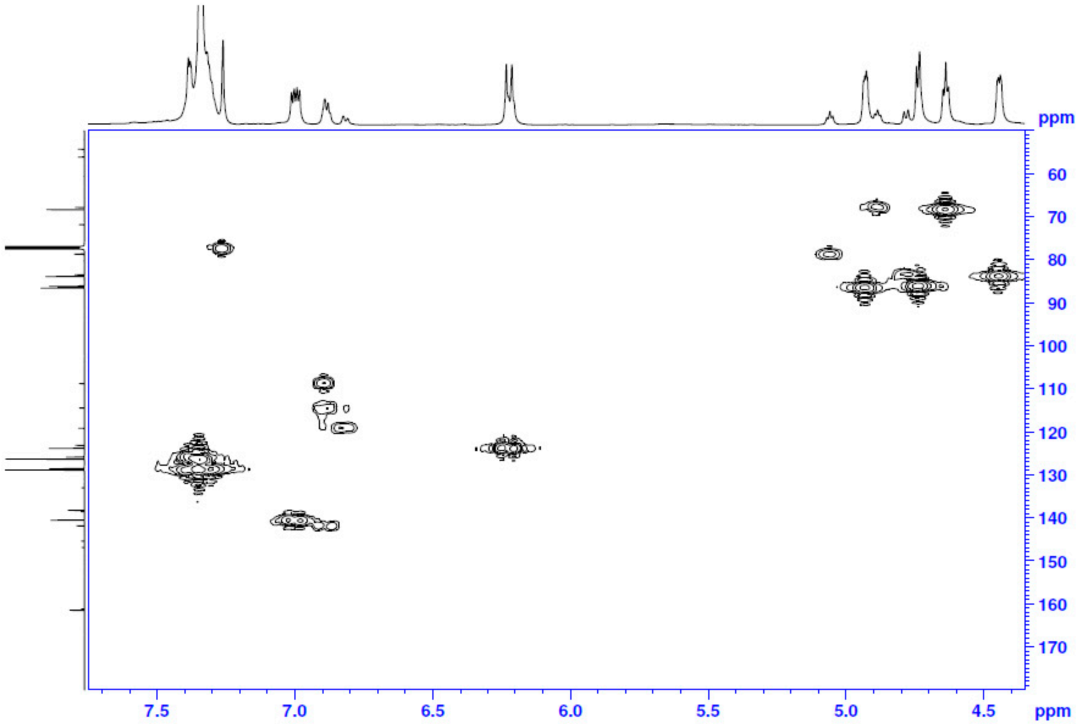

Figure S5 Expansion of HMQC spectrum of **1** in $CDCl_3$

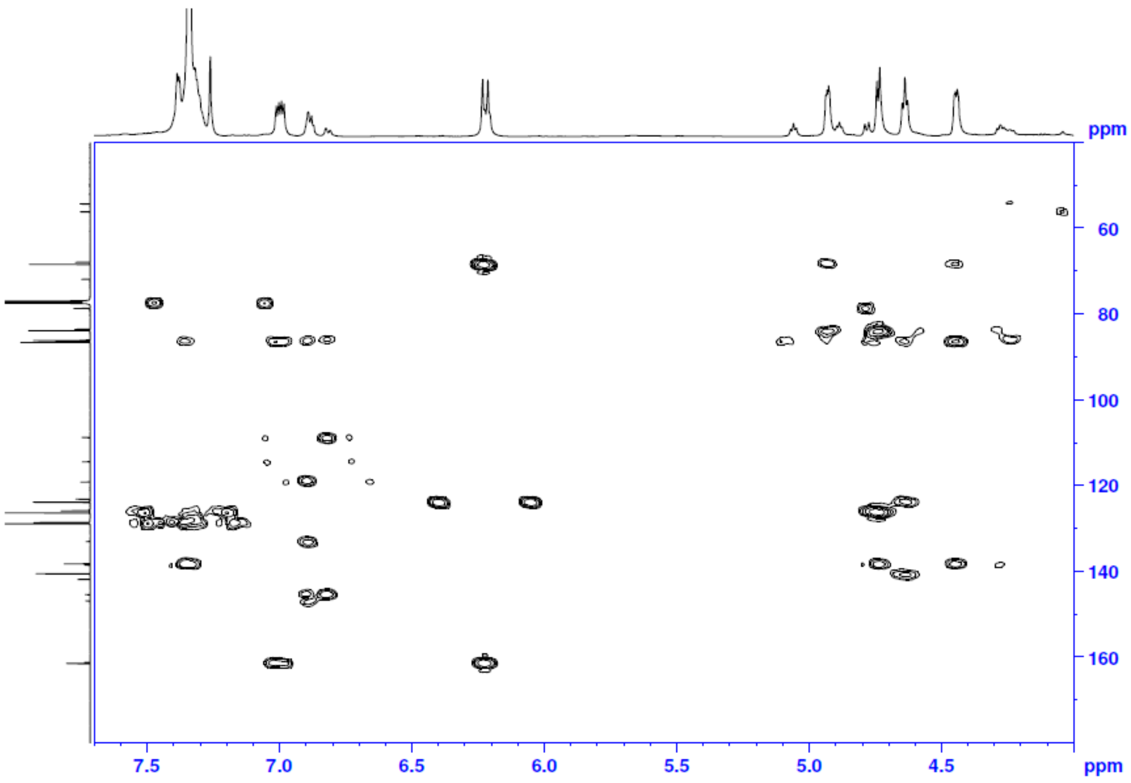

Figure S6 Expansion of HMBC spectrum of **1** in $CDCl_3$

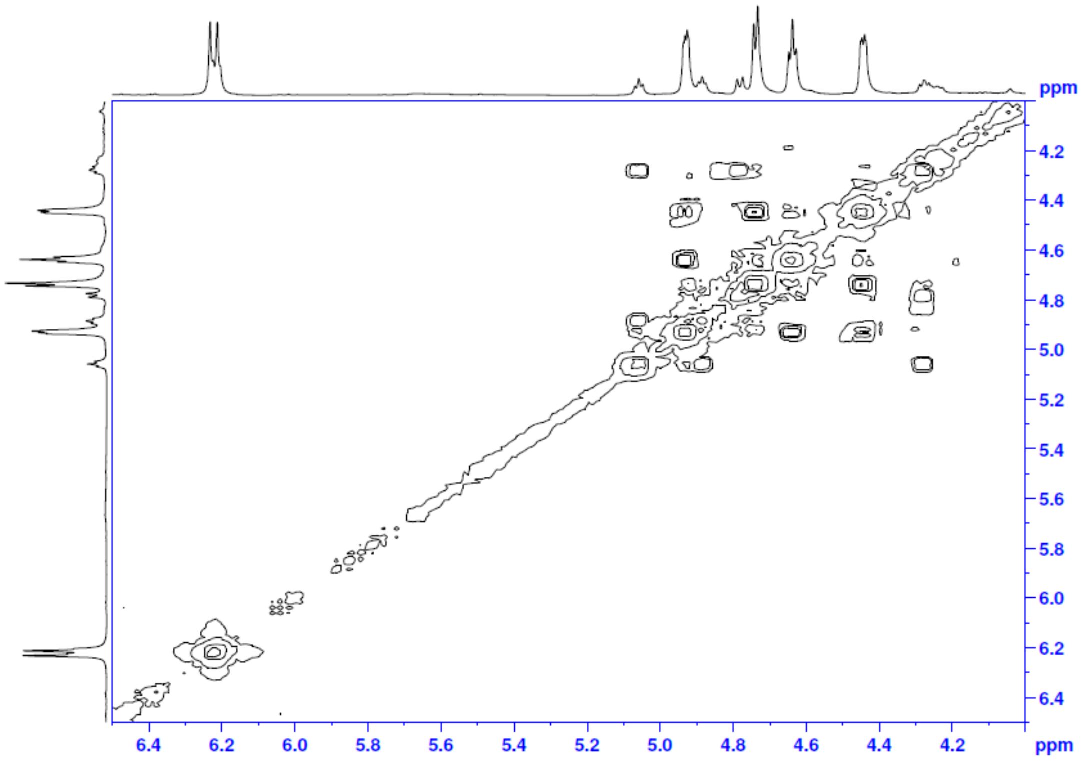

Figure S7 Expansion of COSY spectrum of **1** in $CDCl_3$

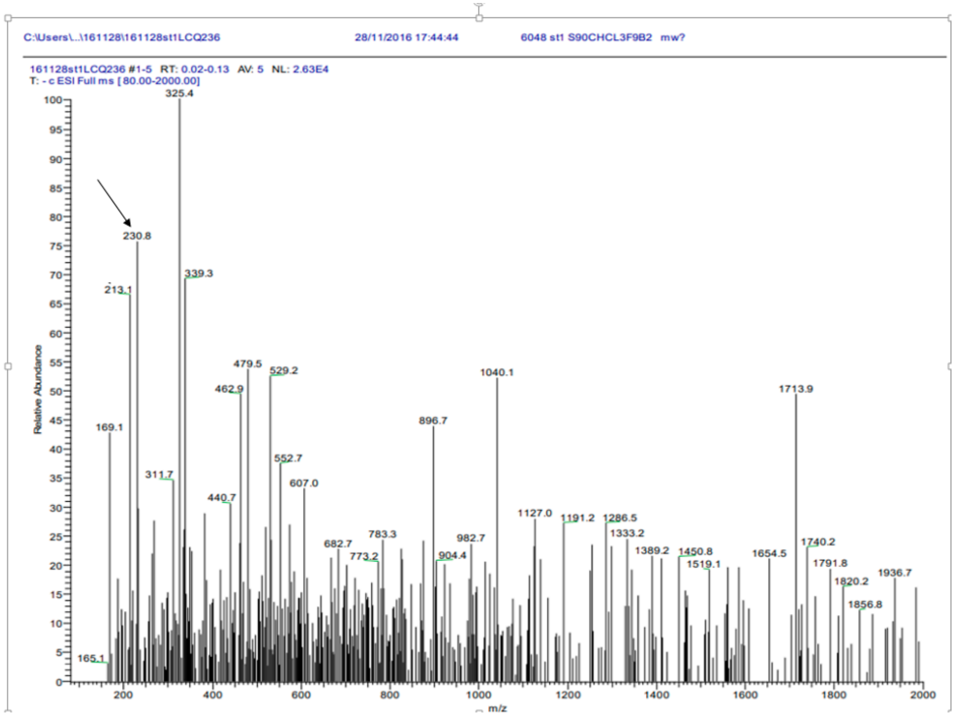

Figure S8 ESI-MS spectrum of **1** showing peak ion at *m/z* 238.1 [M-H] (indicated by arrow)

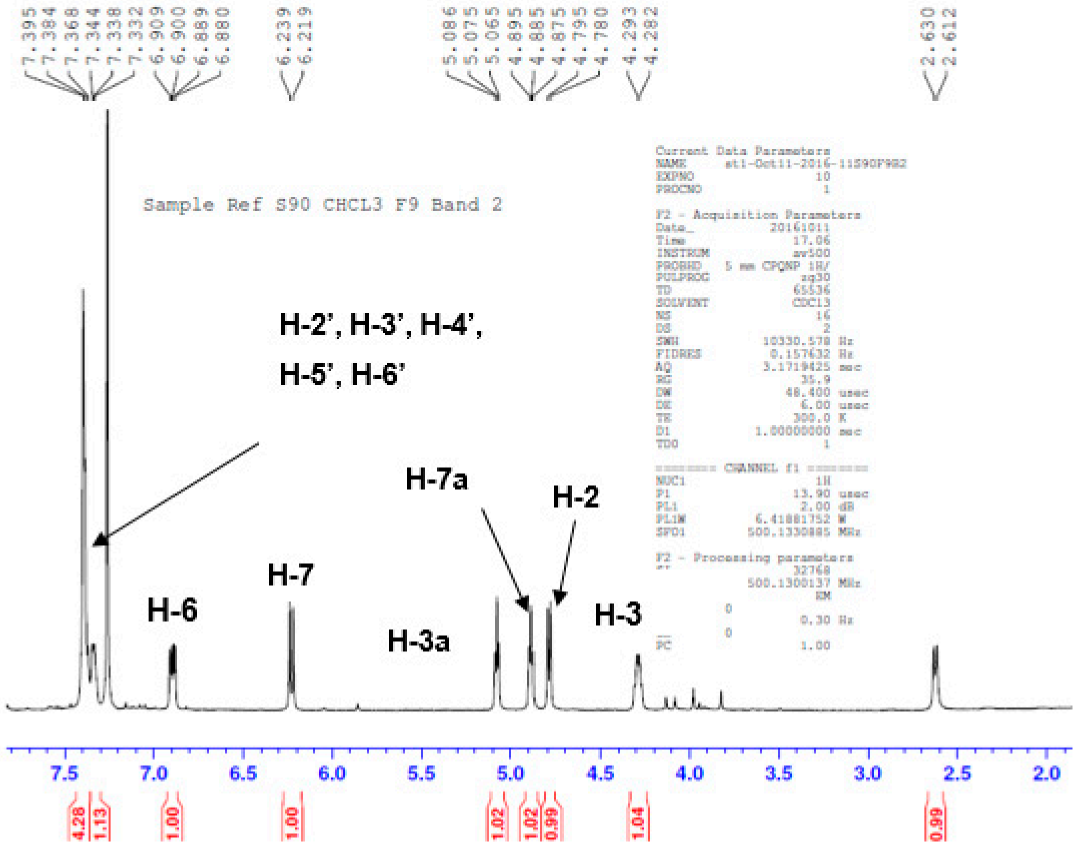

Figure S9 ^{1}H NMR spectrum of **2** in $CDCl_3$ (500 MHz)

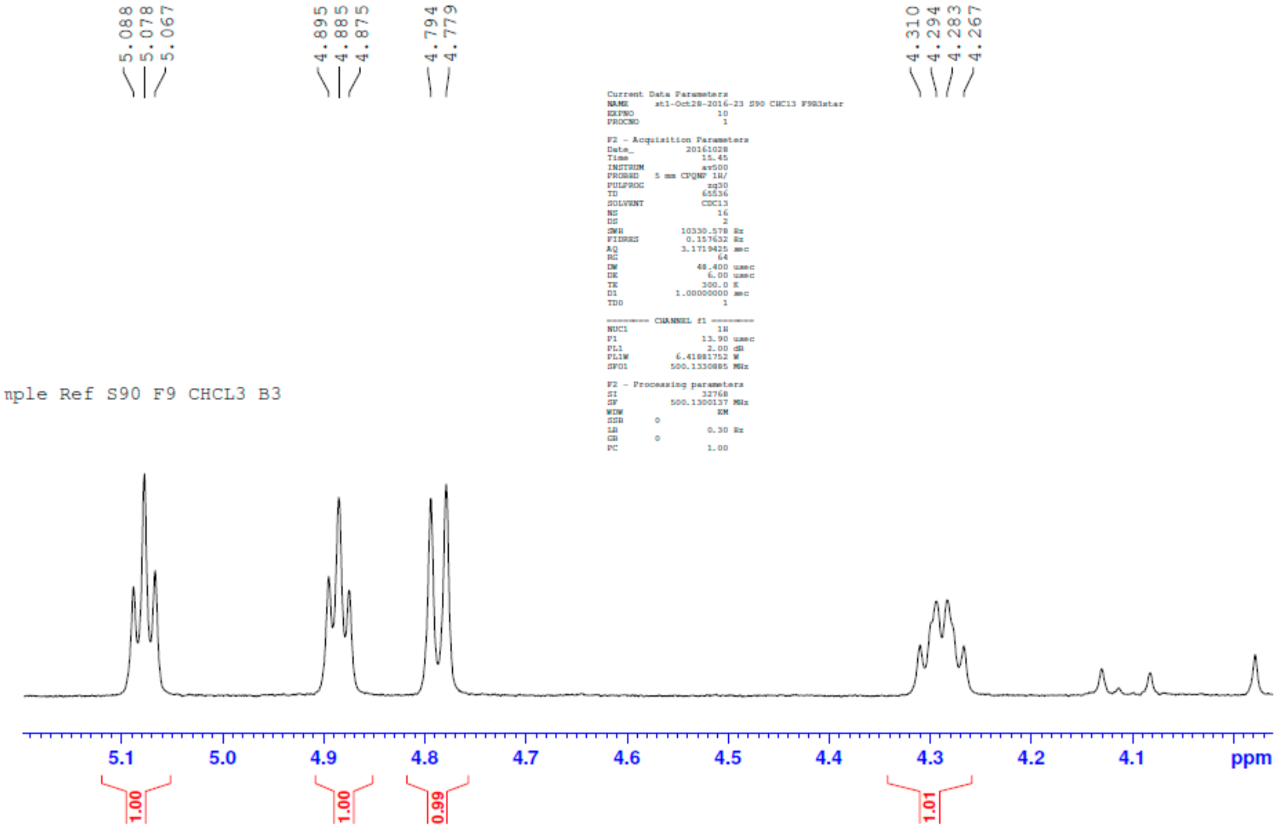

Figure S10 ^{1}H NMR spectrum of **2** in $CDCl_3$ (500 MHz)

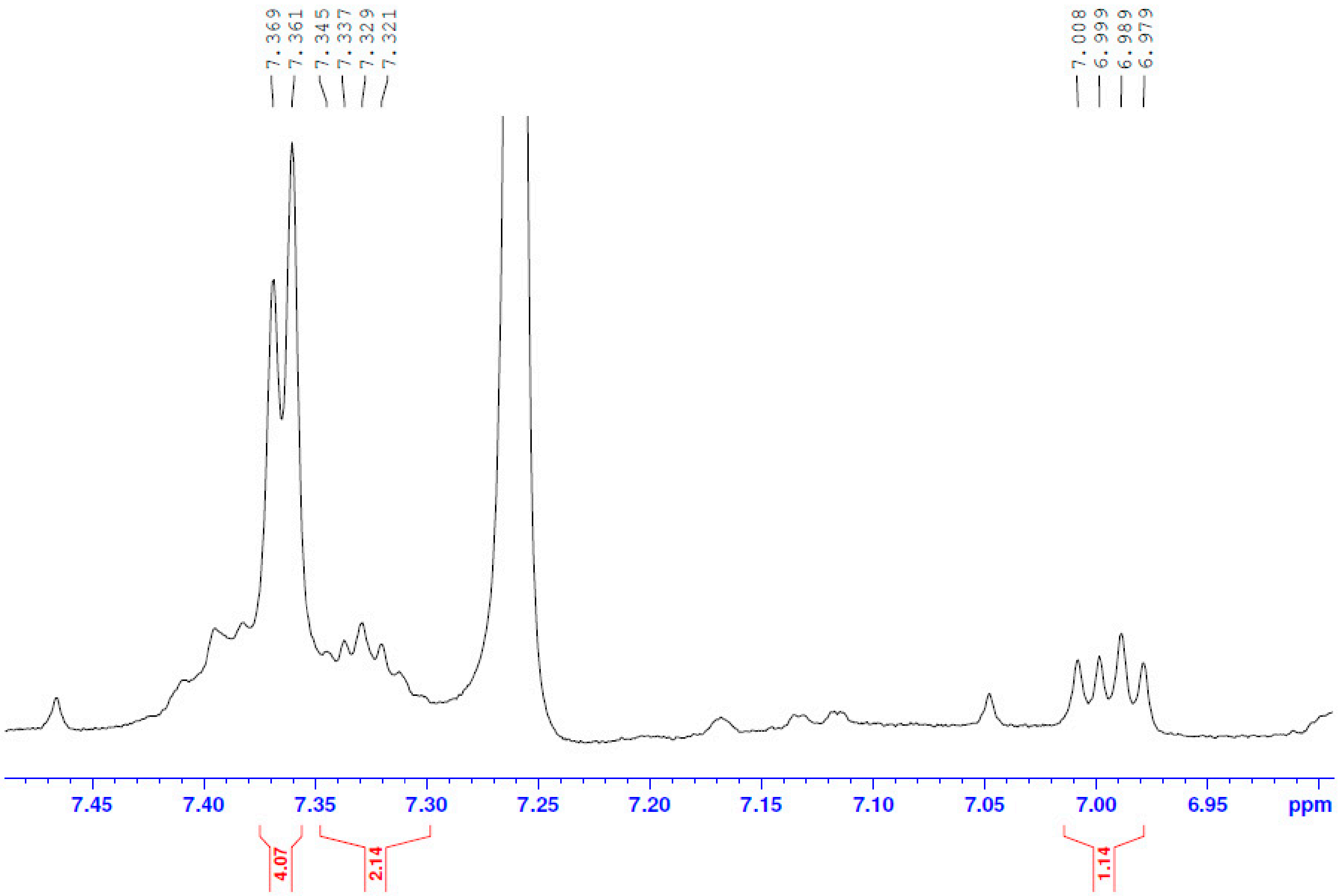

Figure S11 ^{1}H NMR spectrum of **2** in $CDCl_3$ (500 MHz)

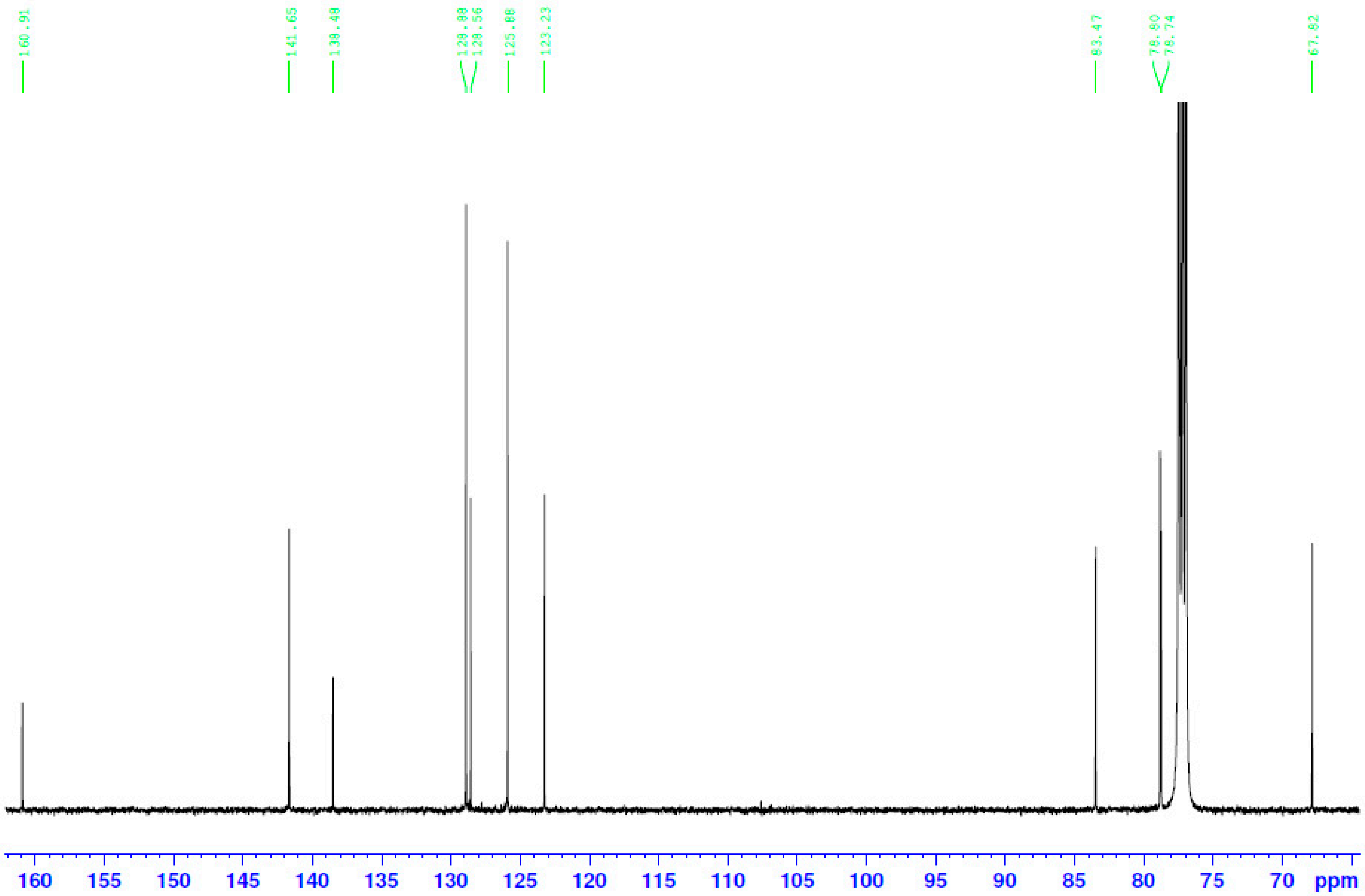

Figure S12 ^{13}CNMR spectrum of **2** in $CDCl_3$ (125 MHz)

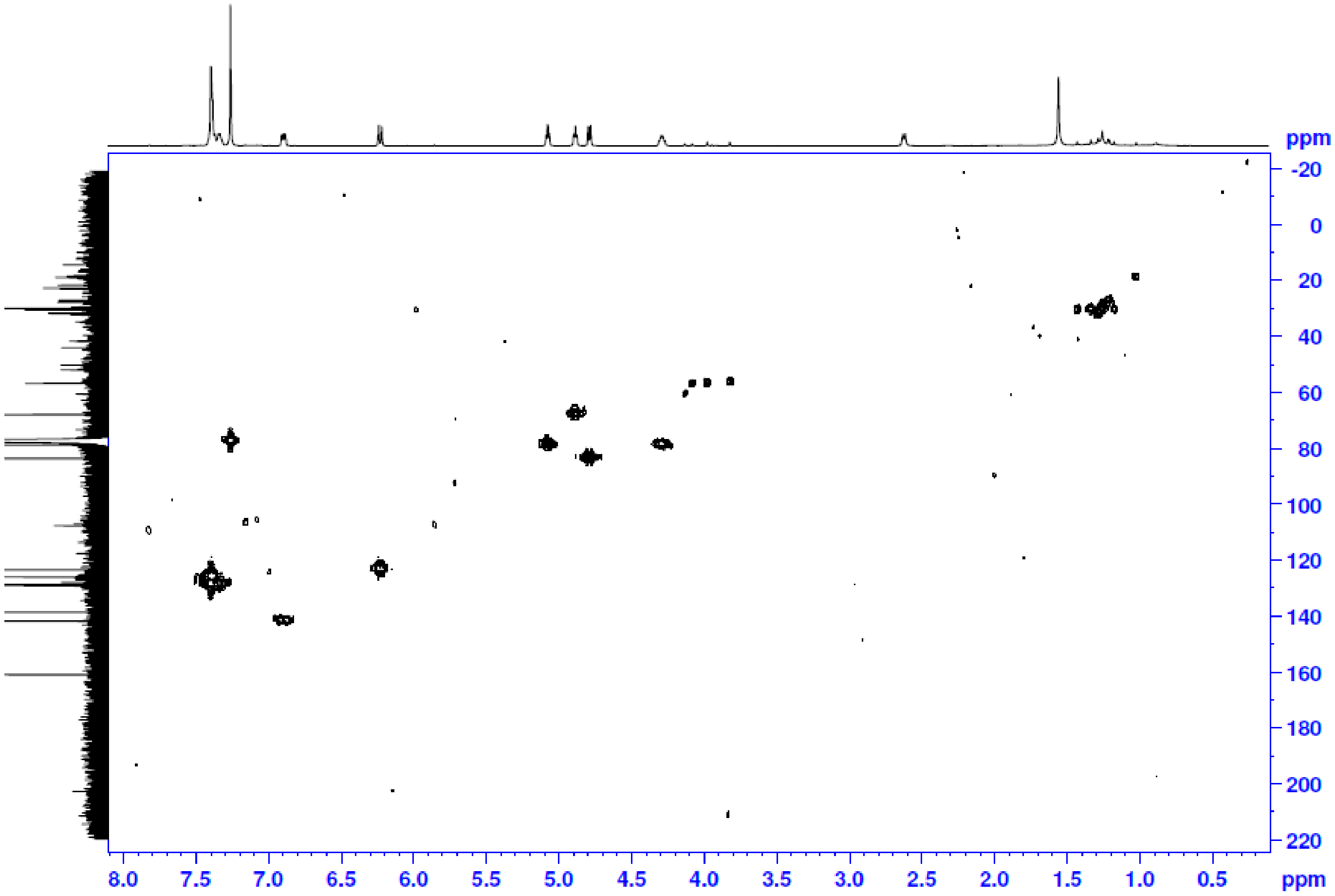

Figure S13 HMQC spectrum of **2** in $CDCl_3$

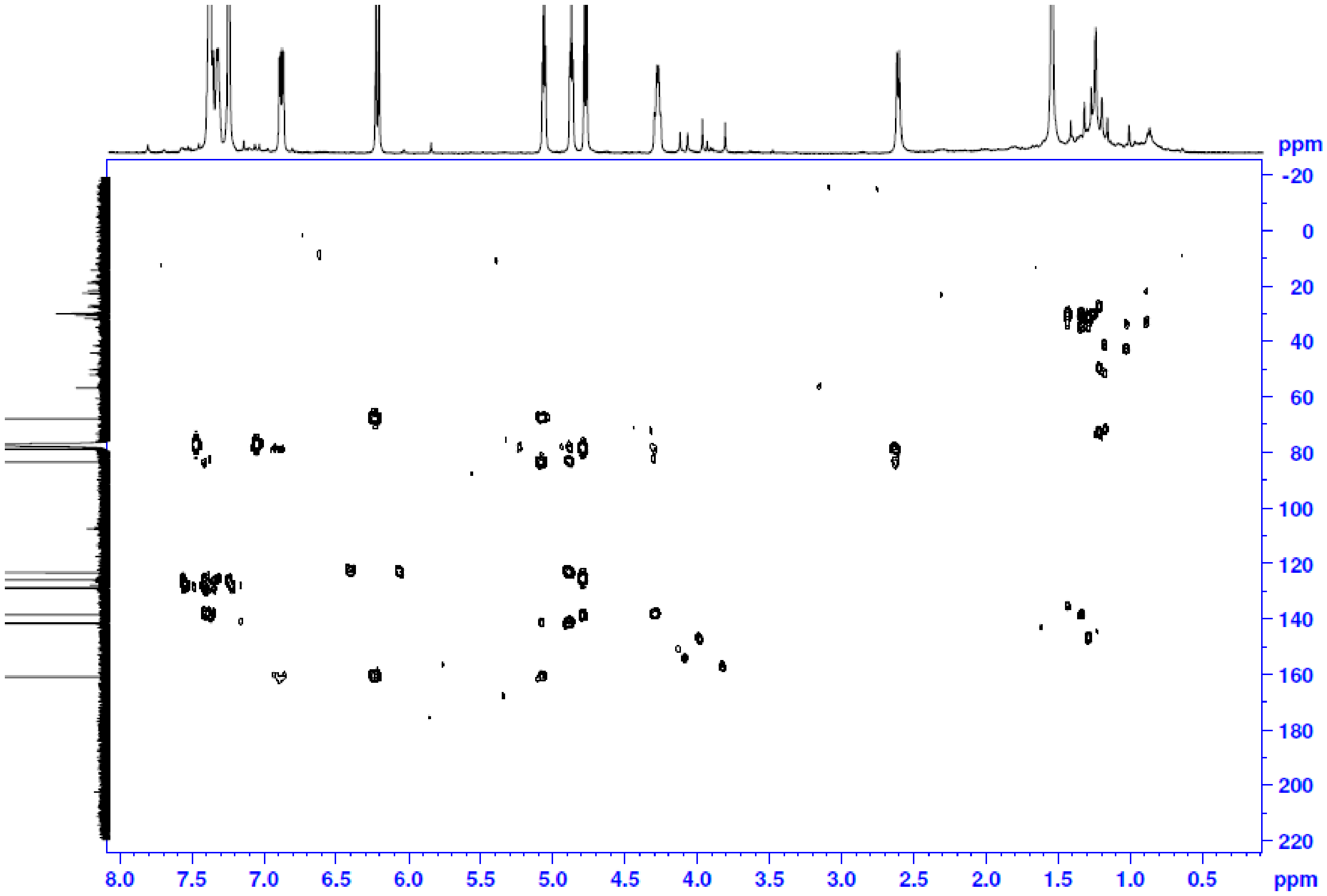

Figure S14 HMBC spectrum of **2** in $CDCl_3$

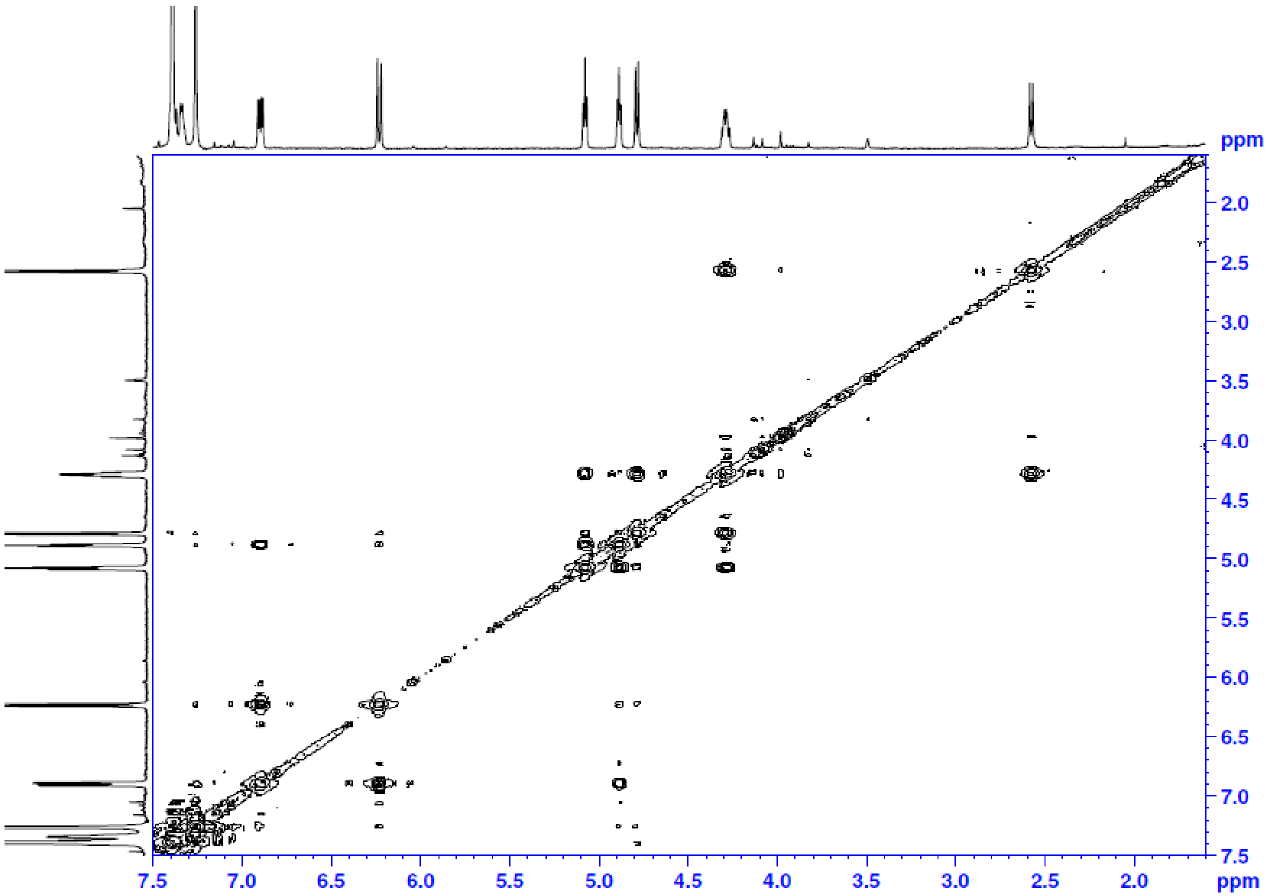

Figure S15 COSY spectrum of **2** in $CDCl_3$

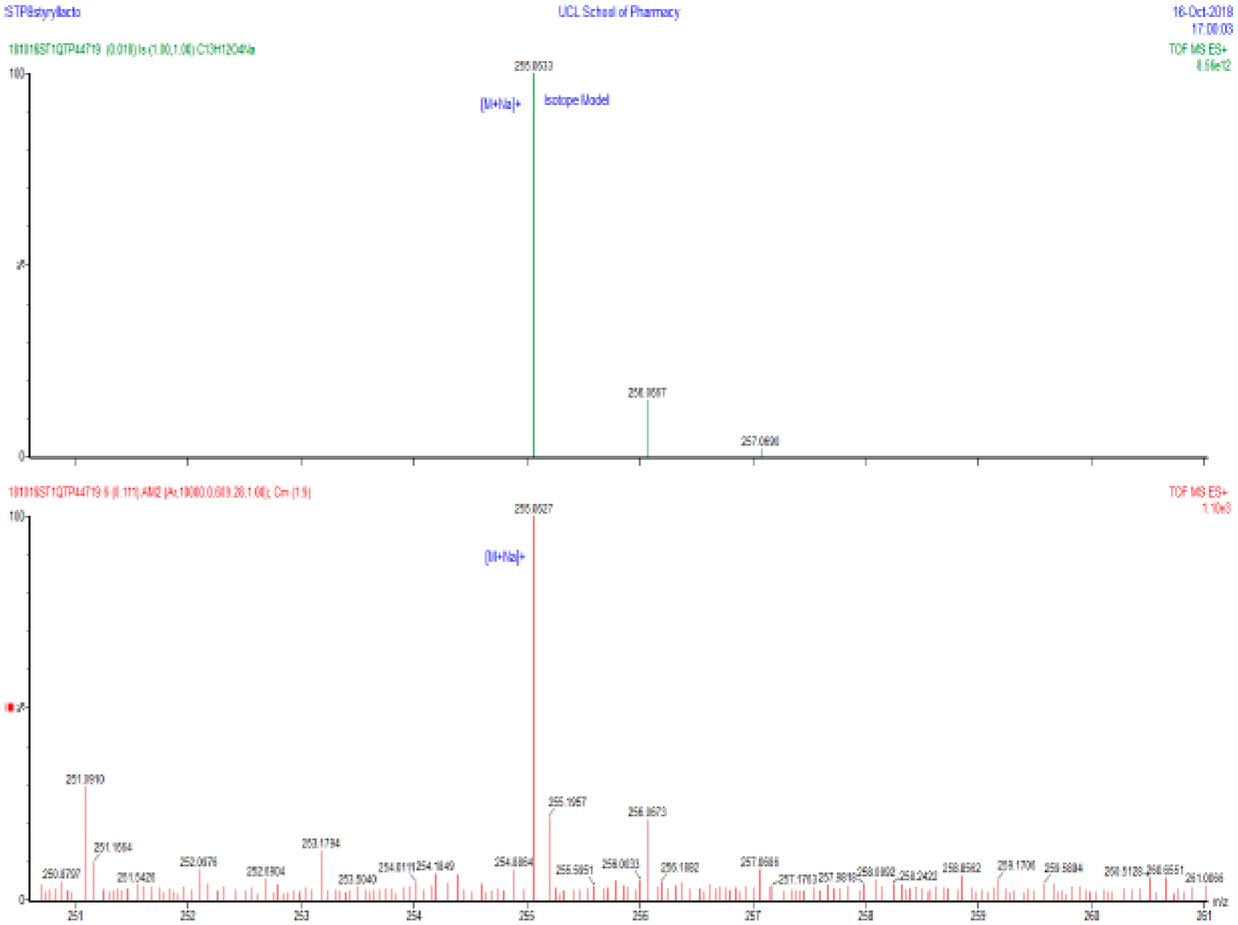

Figure S16 HRMS spectrum of **2** showing peak ion at *m*/*z* 255.0627.

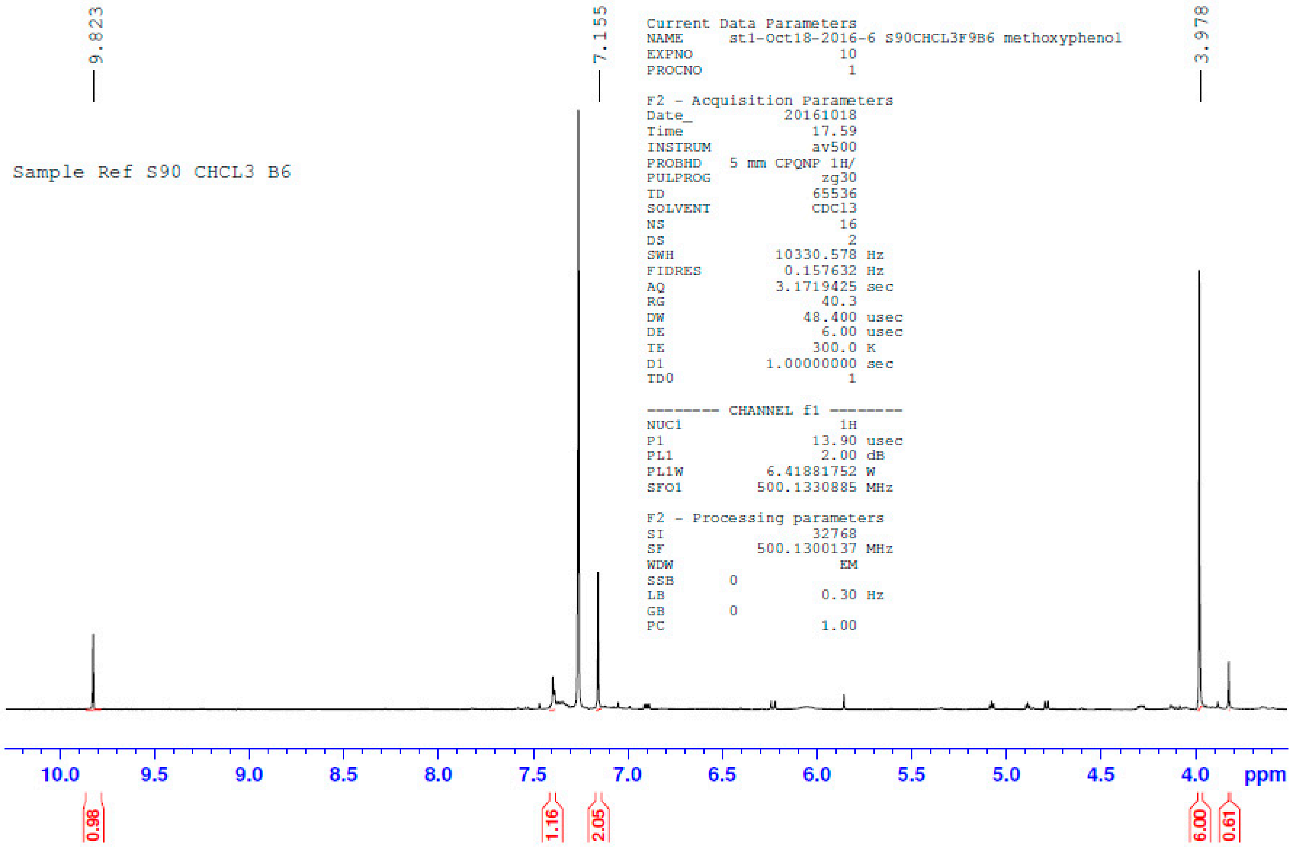

Figure S17 ^{1}H NMR spectrum of **3** in $CDCl_3$ (500 MHz)

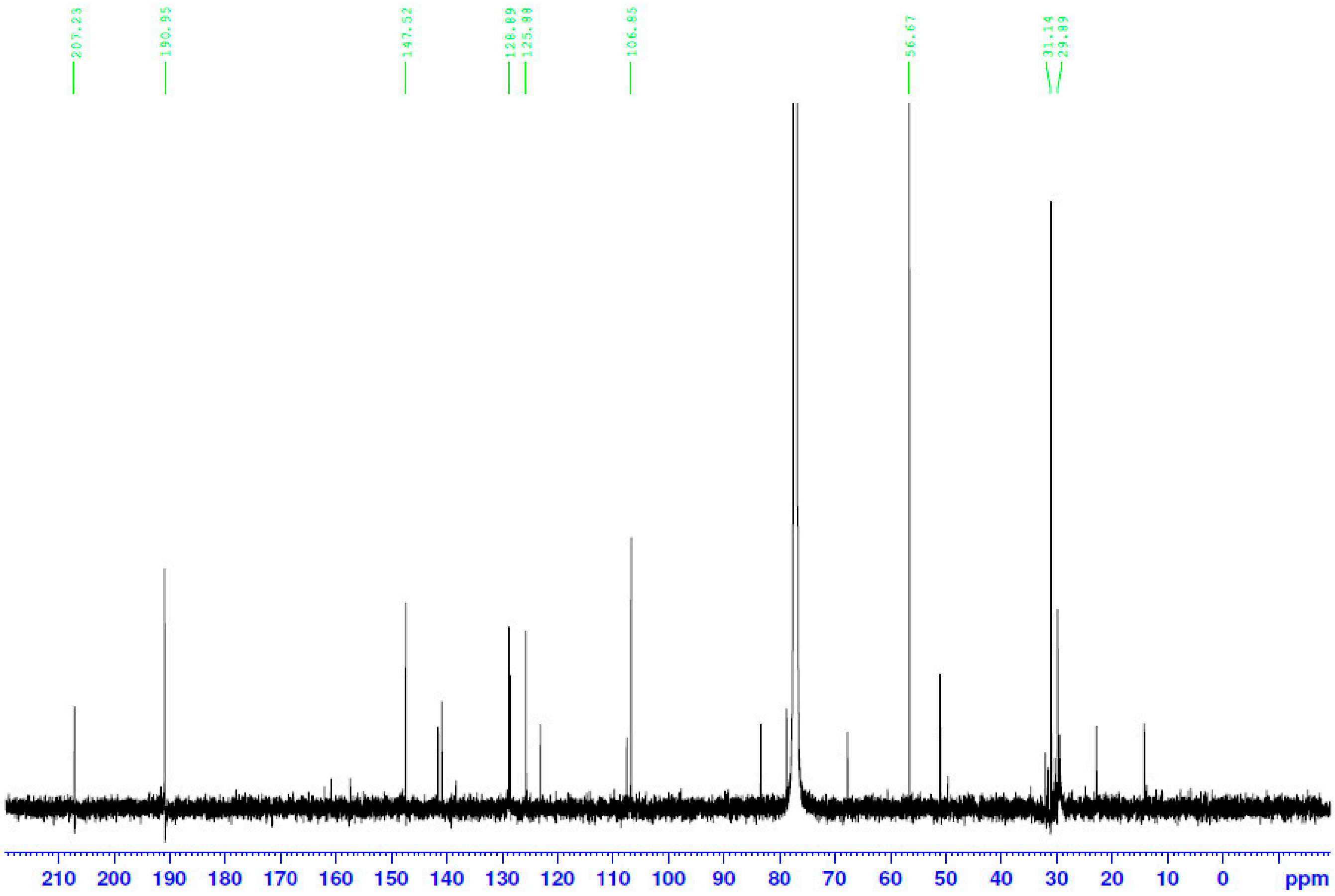

Figure S18 ^{13}C spectrum of **3** in $CDCl_3$ (125 MHz)

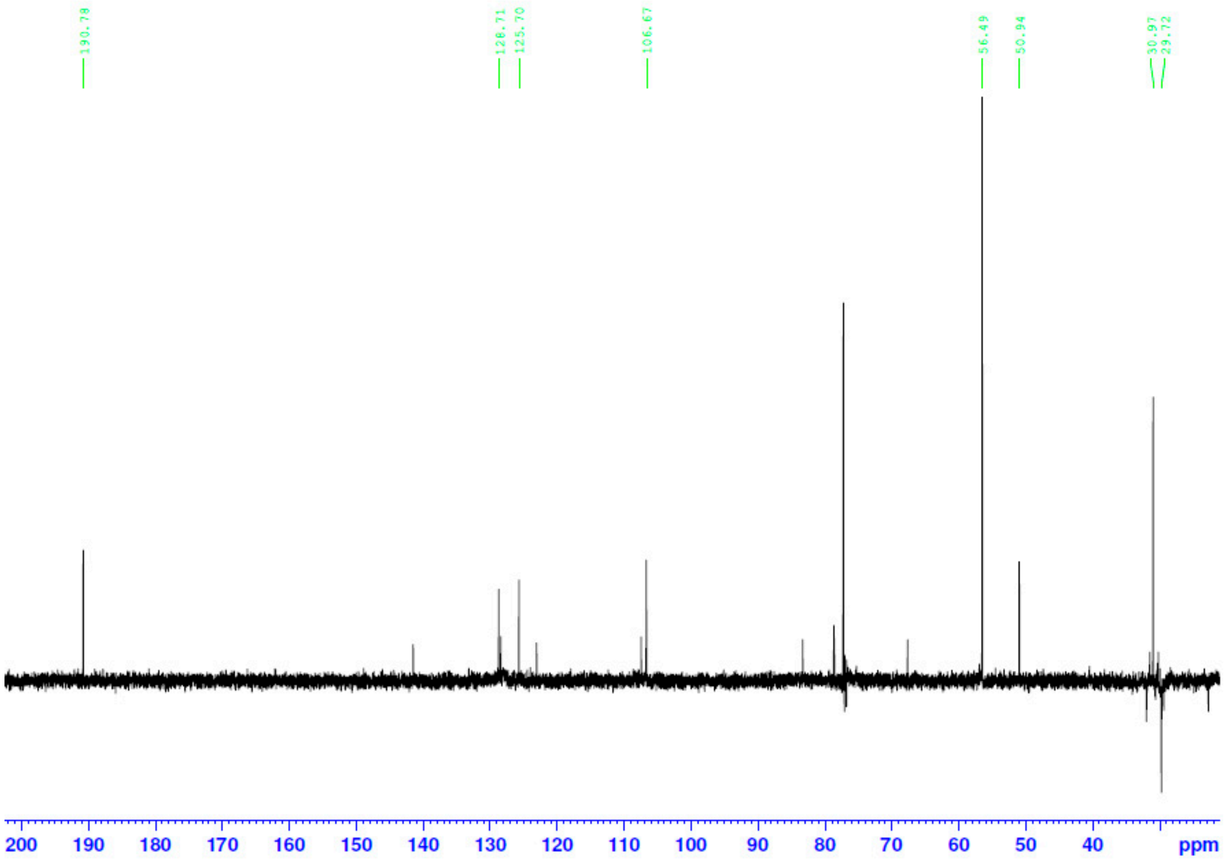

Figure S19 ^{13}C spectrum of **3** in $CDCl_3$ (125 MHz).

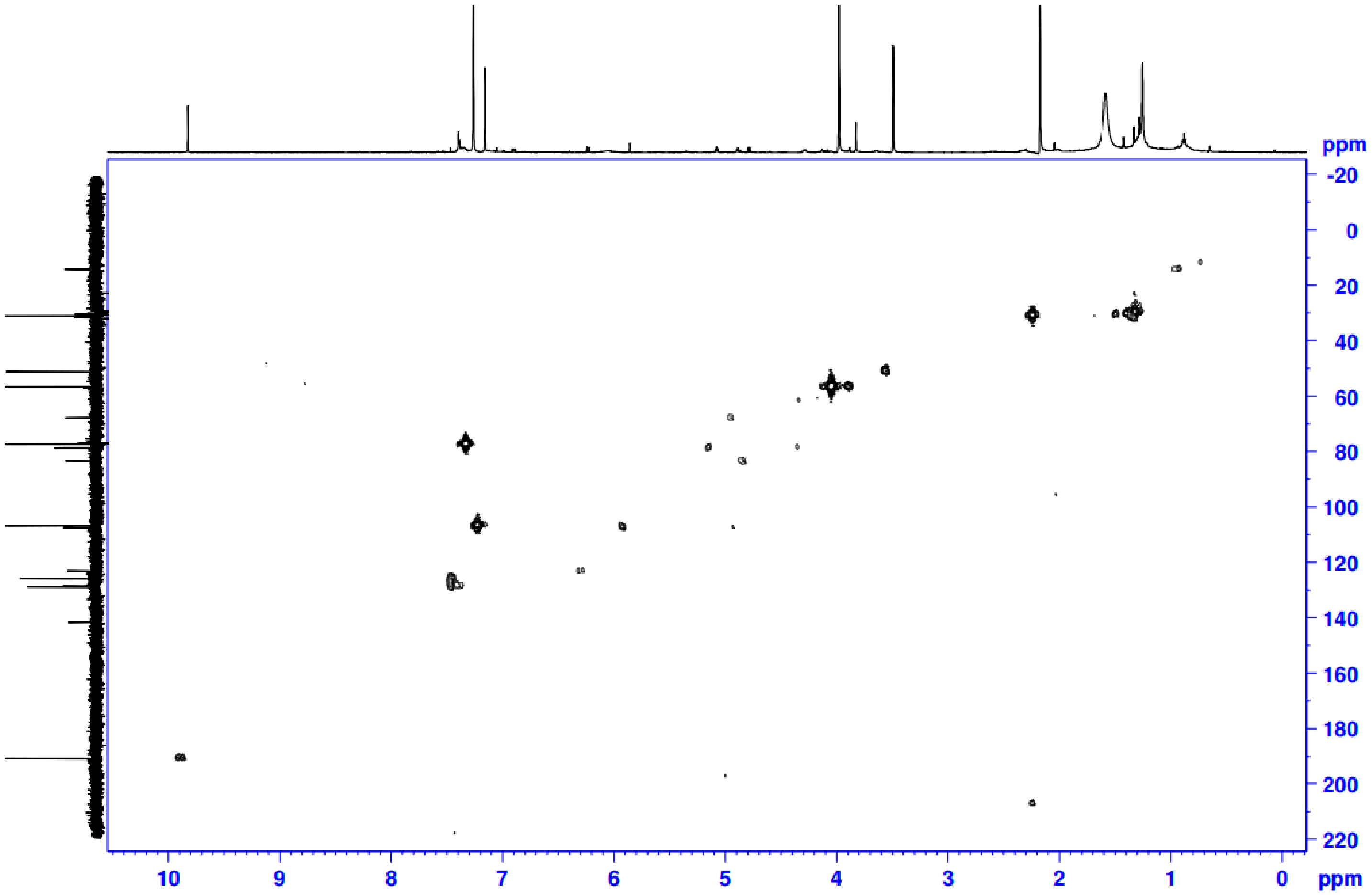

Figure S20 HMQC spectrum of **3** in $CDCl_3$

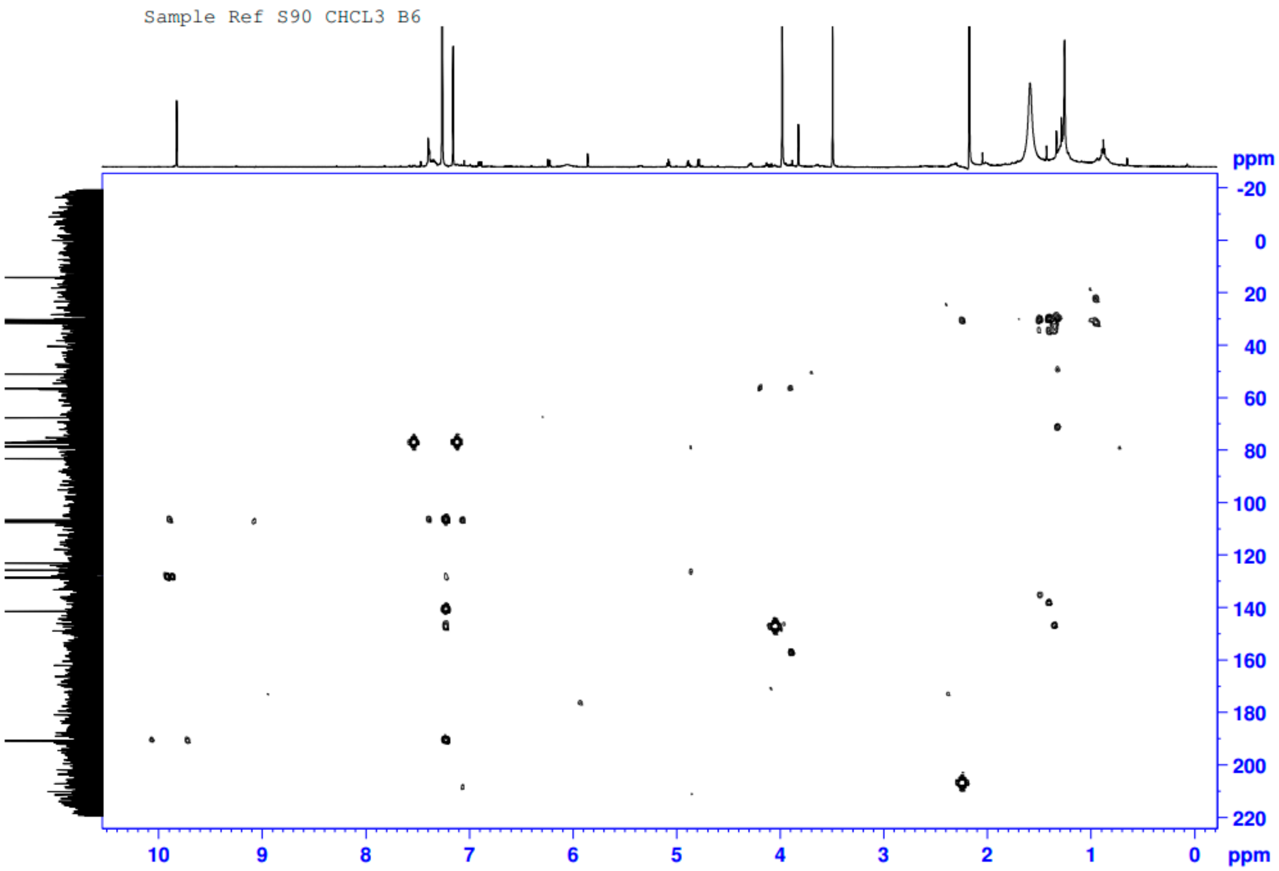

Figure S21 HMBC spectrum of **3** in $CDCl_3$

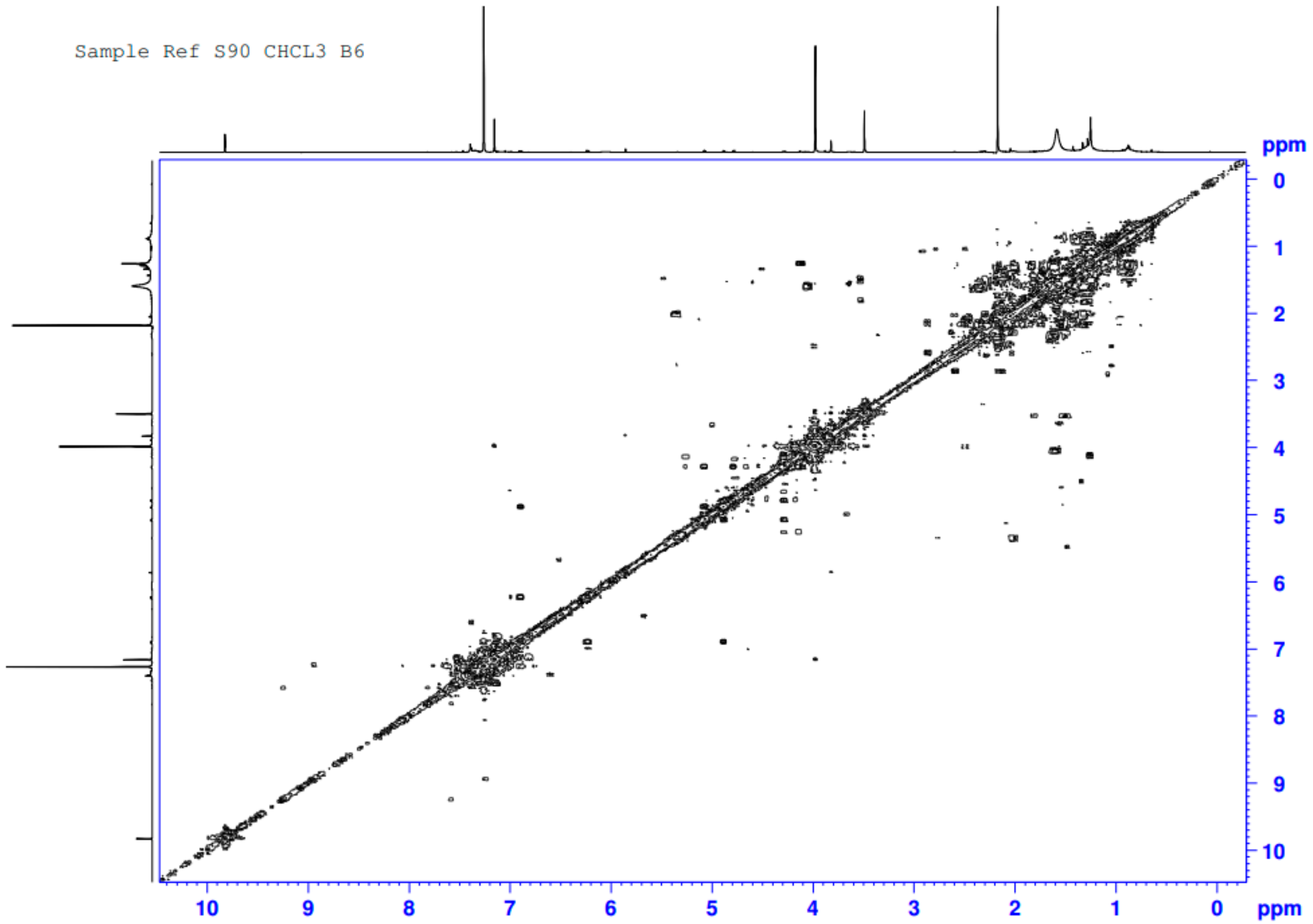

Figure S22 COSY spectrum of **3** in $CDCl_3$

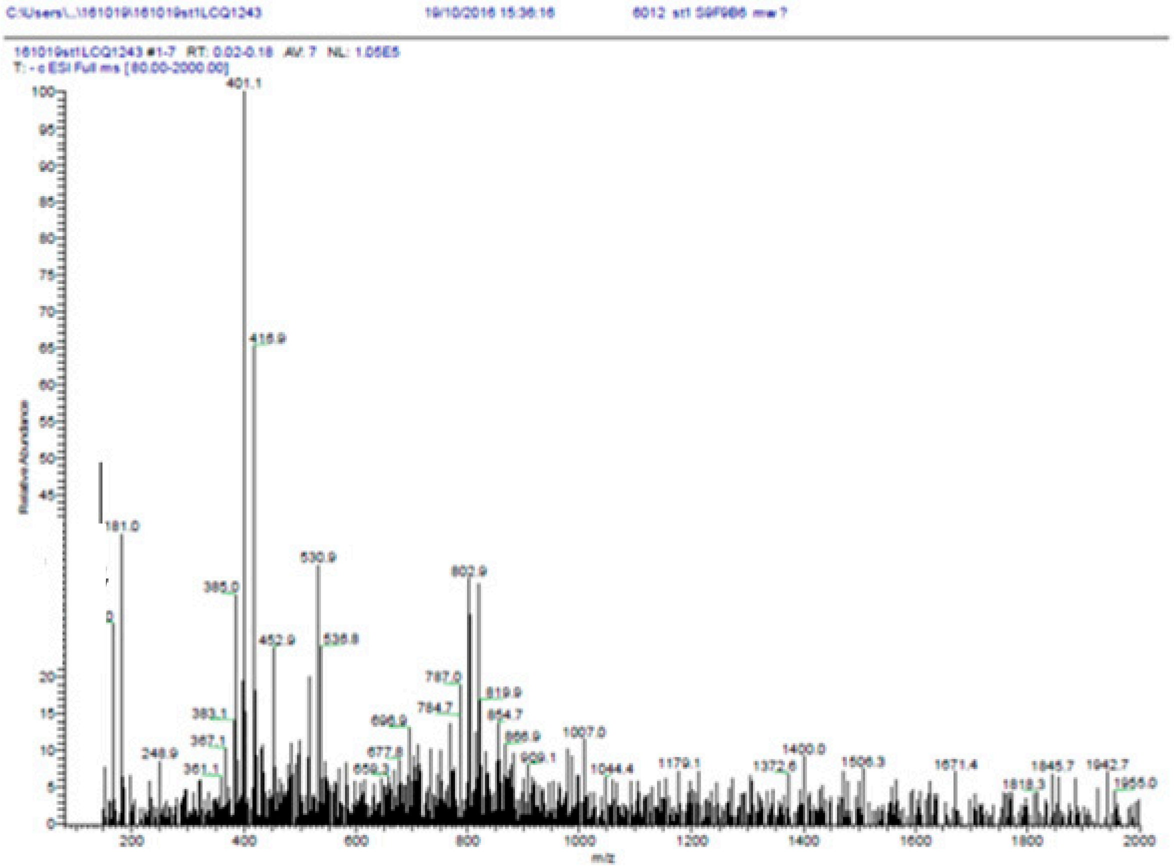

Figure S23 ESI-MS spectrum of **3** showing peak ion at *m/z* 248.9 (indicated by arrow)

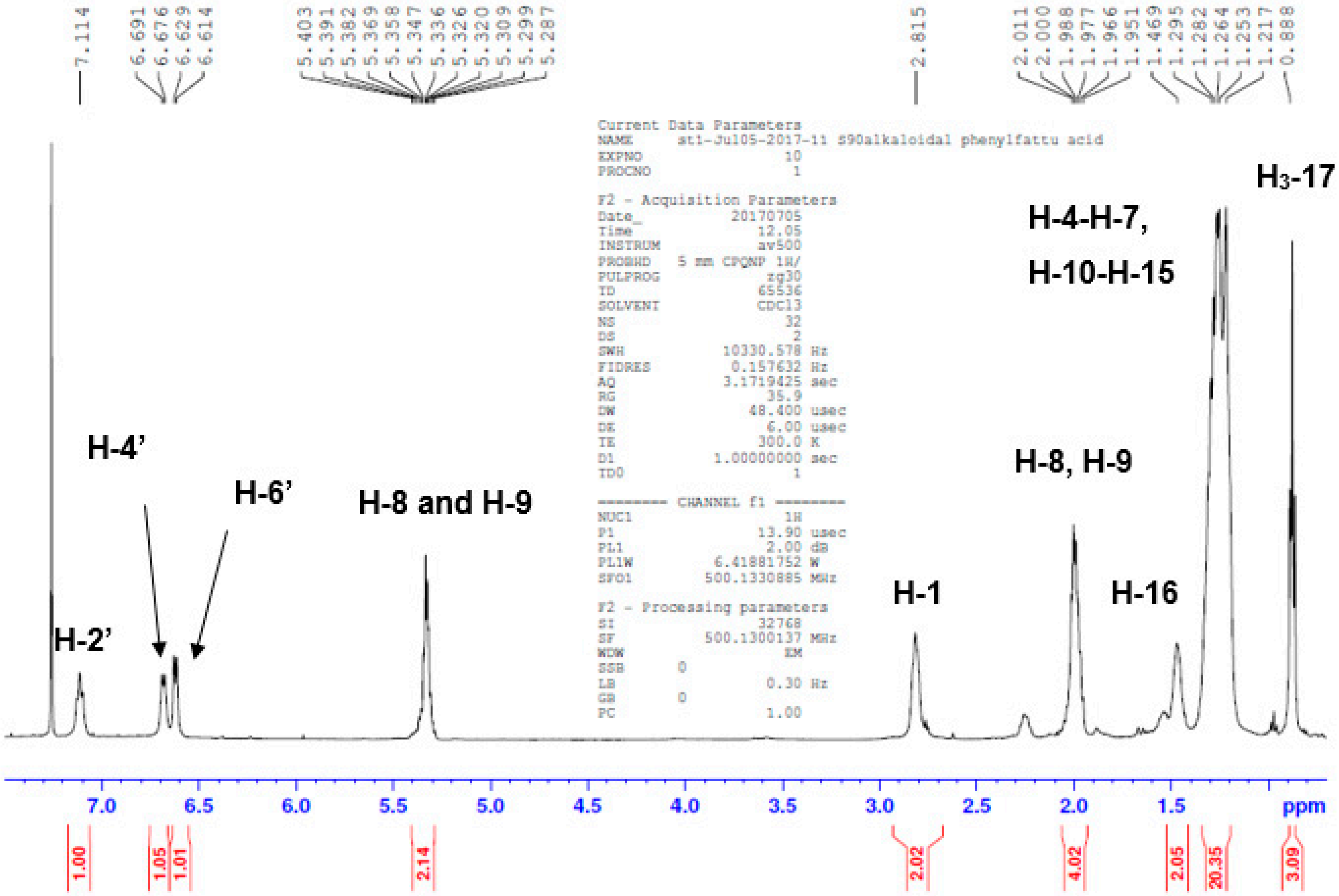

Figure S24 ^{1}H NMR spectrum of **4** in $CDCl_3$ (500 MHz)

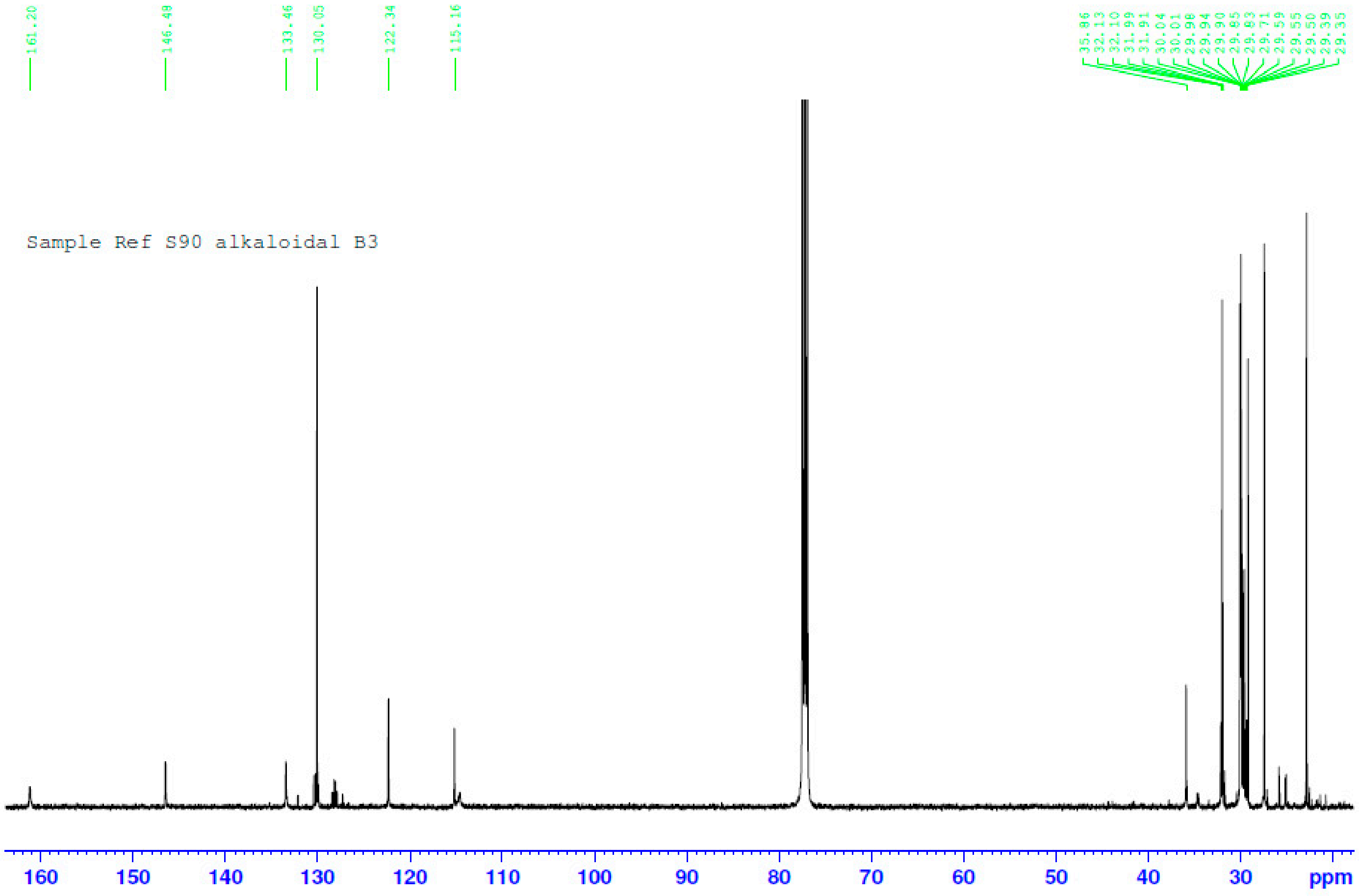

Figure S25 ^{13}C NMR spectrum of **4** in $CDCl_3$ (125 MHz)

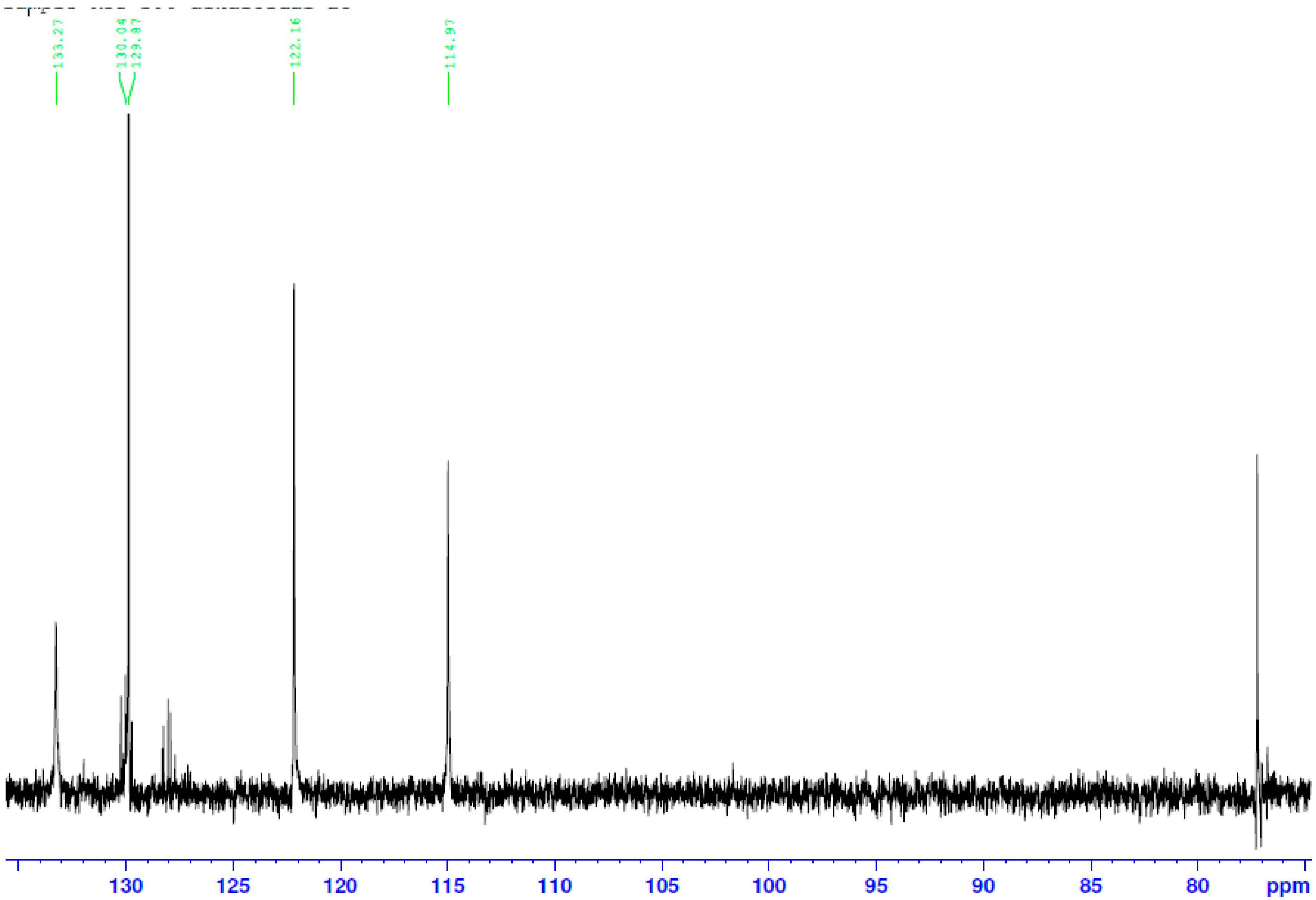

Figure S26 ^{13}C NMR spectrum of **4** (expanded) in $CDCl_3$ (125 MHz)

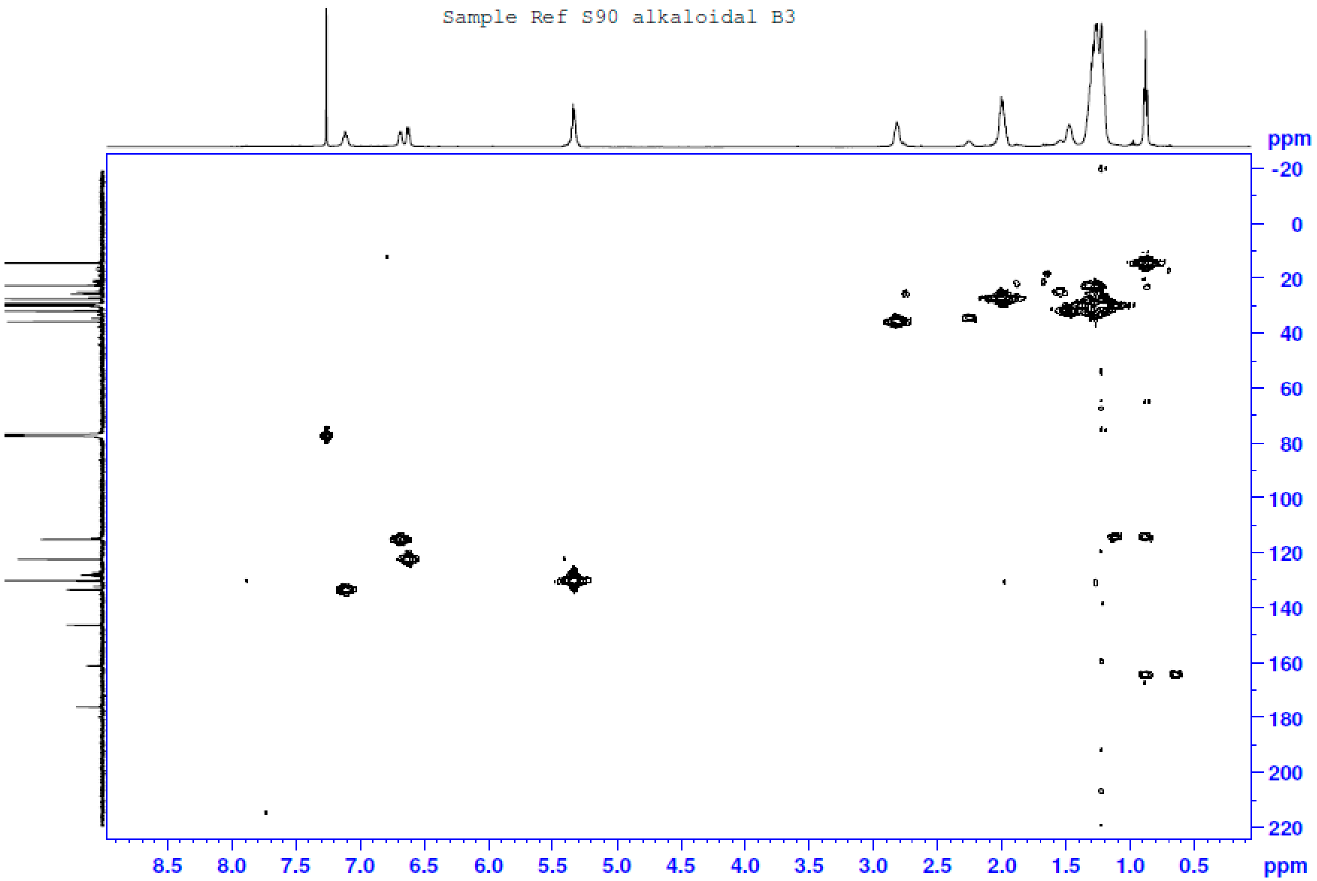

Figure S27 HMQC spectrum of **4** in $CDCl_3$

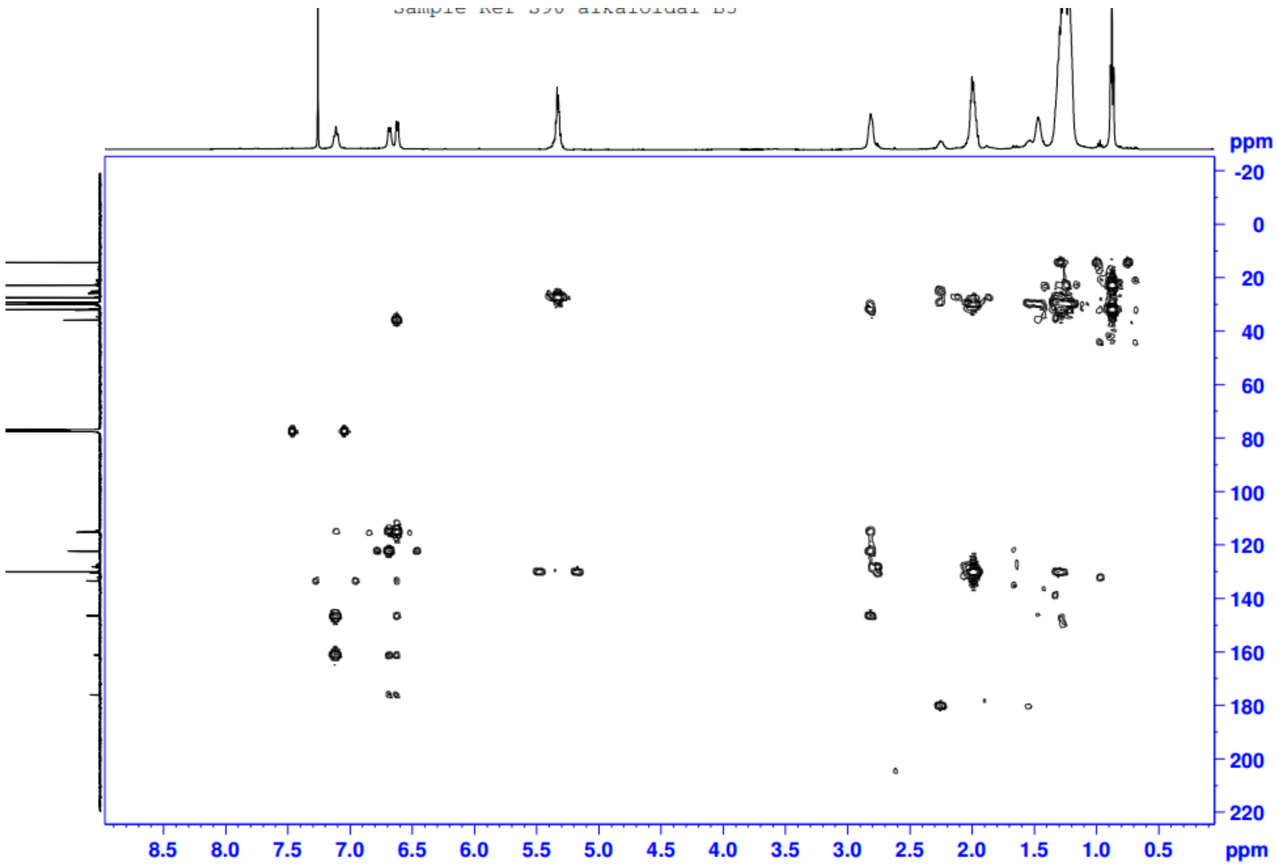

Figure S28 HMBC spectrum of **4** in $CDCl_3$

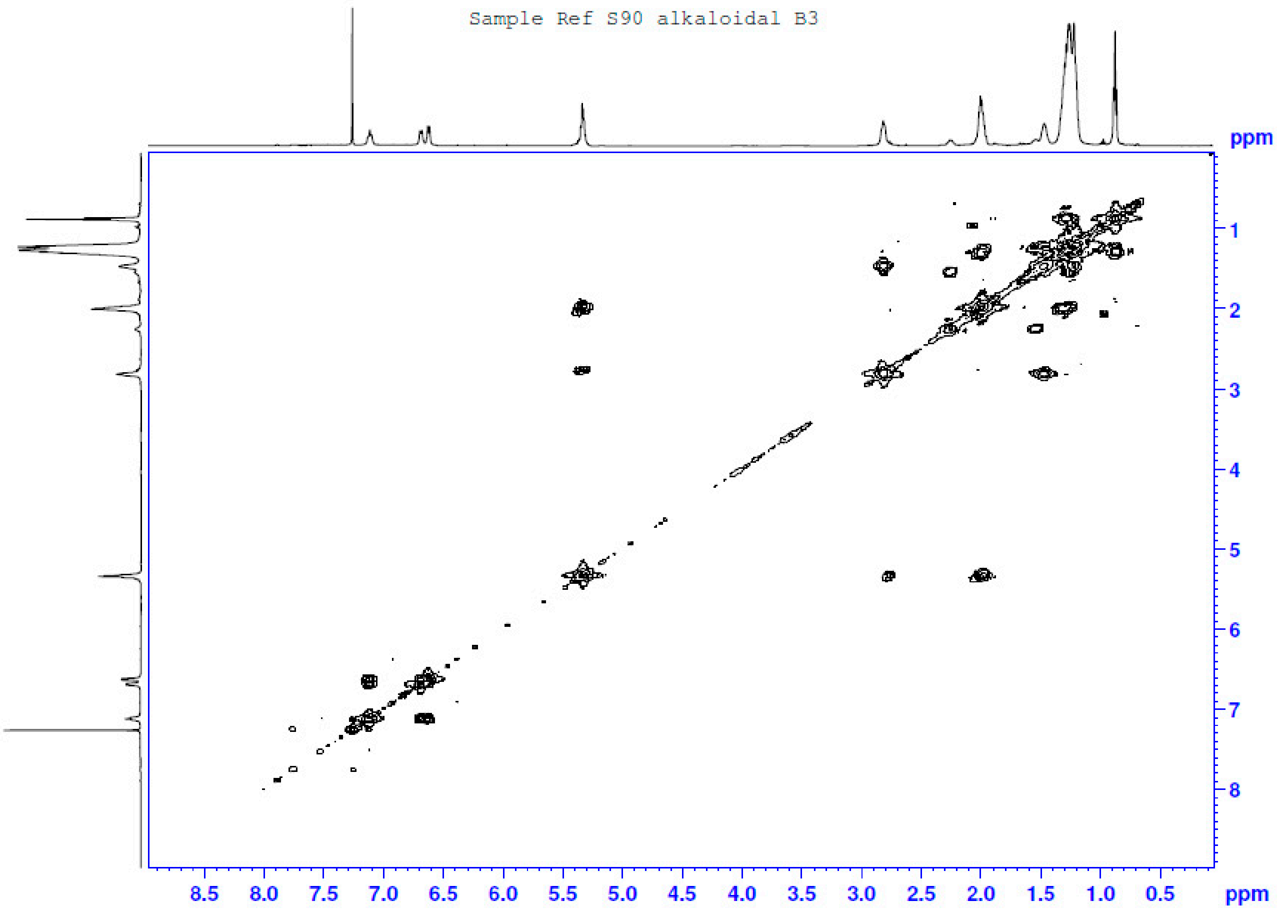

Figure S29 COSY spectrum of **4** in $CDCl_3$

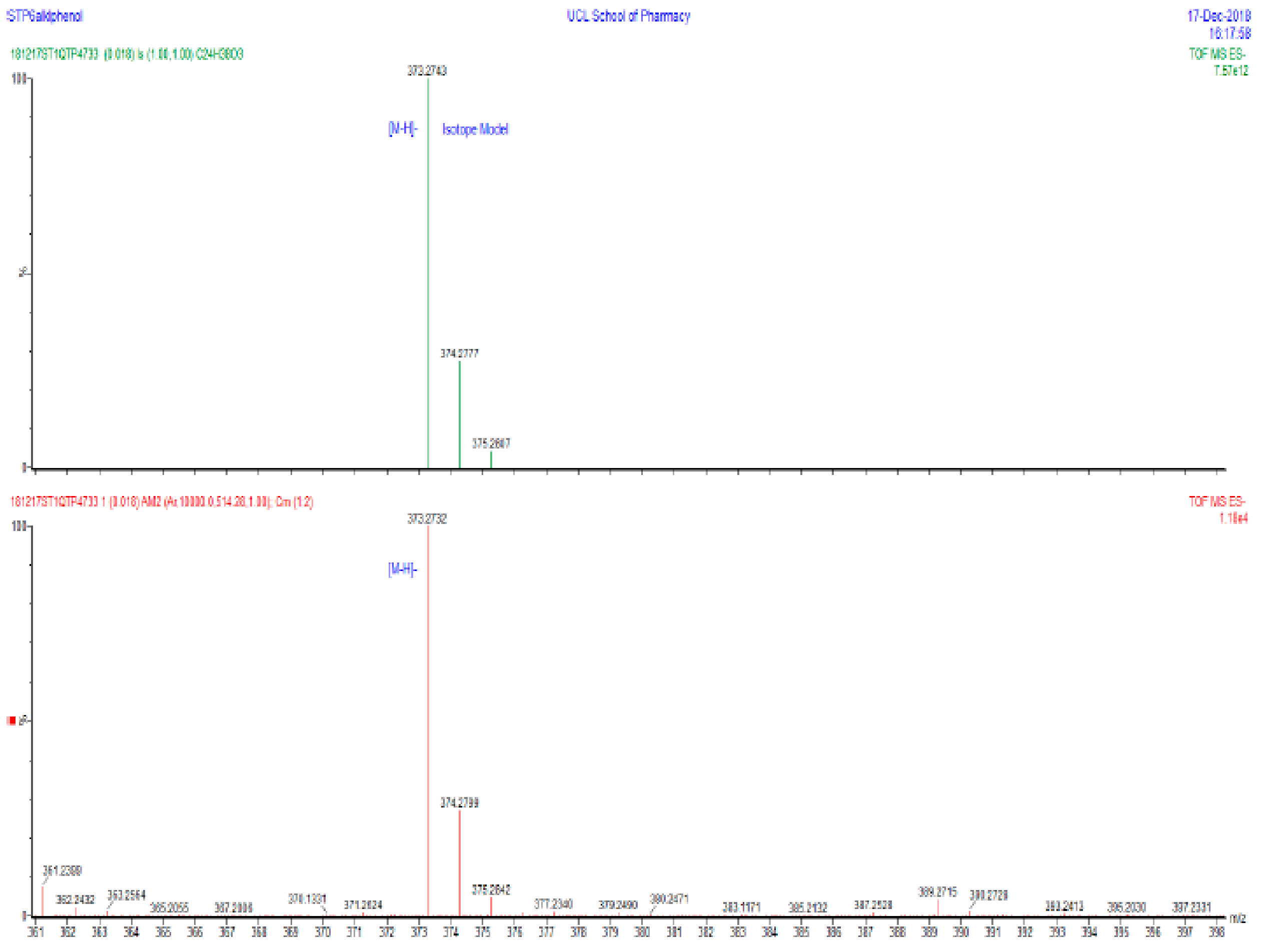

Figure S30 HRMS spectrum of **4** showing peak ion at *m*/*z* 373.2732

1.0 *Phytochemistry*

Compound **1** is the known styryllactone (+)-altholactone, also known as goniothalenol ((2*S*,3*R*,3a*S*,7a*S*)-3-hydroxy-2-phenyl-2,3,3a,7a-tetrahydrobenzo-5(4*H*)-5-one). The molecular formula was determined as $C_{13}H_{12}O_4$ and the molecular weight of the compound was calculated as 232.07, which corresponded to an ion in the MS at *m/z* 231 $[M-H]^-$. The ^1H and ^{13}C NMR data of **1** were in close agreement for those of (+)-altholactone (**Table S1** and **S2**) [1]. The stereochemistry is determined by the size of the vicinal coupling constant between the hydrogens on the chiral centres and compared that with the 6-membered ring compound model provided by Reich (2020) [3] to ascertain their spatial orientation. There are 4 chiral centre for the styryllactone, C-2, C-3, C3a and C-7a. The stereochemistry of **1** was also determined by analysis and comparison of its hydrogen vicinal couplings with the literature for (+)-altholactone [1]. Consequently, compound **1** was assigned as the styryllactone (+)-altholactone((2*S*,3*R*,3a*S*,7a*S*)-3-hydroxy-2-phenyl-2,3,3a,7a-tetrahydrobenzo-5(4*H*)-5-one).

Compound **2** was proposed as a new styryllactone, (2*S*,3*R*,3a*S*,7a*R*)-3-hydroxy-2-phenyl-2,3,3a,7a-tetrahydrobenzofuran-5(4*H*)-one), and are different from all other known stereoisomers i.e. the 3 synthesized epimers [4] (one was later isolated as a natural product, (+)-isoaltholactone [2]) in terms of ^{13}C and ^1H chemical shifts (**Table S2**) as well as the coupling constants. It is, however, similar to a known compound (+)-isoaltholactone since the ^{13}C and ^1H chemical shifts were similar [2] but differing in the coupling constant between H-3a and H-7a since the coupling constants for (+)-isoaltholactone and **2** were 5 Hz and 10 Hz respectively, hence the difference in the stereo-orientation for H-3a and H-7a. An ion peak $[M+Na]^+$ at 255.0627 in its HRMS indicated a molecular formula of $C_{13}H_{12}O_4$ (calcd. for $C_{13}H_{12}O_4$ 232.0735). The ^1H NMR spectrum (**Table S2**) showed 11 hydrogens. There were 5 deshielded aromatic hydrogens in the ^1H spectrum. Signals at δ_H 7.34 (C-4′), a further 2 pairs of equivalent hydrogens at δ_H 7.38 (H-2′, H-6′) and δ_H 7.36 (H-3′, H-5′) ppm indicated the presence of a phenyl ring as in **1**. 2 olefinic hydrogens at δ_H 6.90 (H-6) and 6.62 (H-7) ppm were similar to those of **1** and attributable to the olefin of the pyranone ring system. Couplings in the COSY spectrum between δ_H 4.29 (H-3), 4.79 (H-2), 5.08 (H-3a) and 4.88 (H-7a) ppm could also be identified, with H-7a coupling to H-7. A hydroxy group could also be placed at C-3 as in **1** above. The ^{13}C NMR spectrum (**Table S2**) confirmed the similarities between **1** and **2** and showed 13 carbons, 6 of which are aromatic with signals appearing at δ_C 128.7 (C-1′), 129.0 (C-2′/C-6′), 125.9 ppm (C-3′/C-5′) and 123.9 (C-4′) ppm. As with **1**, there was a 7-carbon cyclic structure (furo-pyranone) attached to the phenyl ring – one of the carbons was a carbonyl at signal δ_C 160.1 (C-5), 2 olefinic carbons at signal δ_C 138.1 (C-6) and 140.3 (C-7) ppm. The other 3 carbons were attached to oxygen with resonances at δ_C 83.5 (C-3a), 78.7 (C-3) and 68.4 (C-7a) ppm, and the final carbon, as in **1** above bore a hydroxyl group (δ 88.7, C-3) ppm.

Table S1 ^1H (500 MHz) and ^{13}C NMR (125 MHz) and HMBC spectroscopic data of **1** recorded in $CDCl_3$ compared with published data

**Carbon**	**^{13}C**	**Hydrogen**	**^1H**	**in D_2O [25]**	
				**^{13}C**	**^1H**
**C-2**	**86.0**	**4.74**	H-2 (d), *J* = 5.5 Hz		4.7, *J* = 5.6 Hz
C-3	83.8	4.45	H-3 (t), *J* = 2.0 Hz, 5.5 Hz		4.4, *J* = 2.2 Hz, 5.6 Hz
C-3a	86.2	4.95	H-3a (t), *J* = 2.0 Hz, 5.5 Hz		4.85, *J* = 2.2 Hz, 5.2 Hz
C-5	161.5				
C-6	140.5	6.23	H-6 (dd,), *J* = 4.5 Hz, 9.5 Hz		6.15, dd, *J* = 10 Hz

C-7	123.6	6.99	H-7 (dd), *J* = 4.0 Hz, 10.0 Hz	6.95 (dd), *J* = 5 Hz, 9.95 Hz
C-7a	68.3	4.67	H7a (t), *J* = 2 Hz, 5 Hz	4.5 (t), *J* = 5.2 Hz
C-1′	138.2			
C-2′ and C-6′	126.3		H-2′, H-6′	
C-3′ and C-5′	128.6	7.31 (m)	H-3′, H-5′	7.3
C-4′	128.8		H-4′,	

Table S2. Comparison of the hydrogen chemical shifts and coupling constants between **2** with 3 other known styryllactones.

^{1}H	Compound 2	(+)-iso altholactone [8]	(+)-7,8-di-epi-Altholactone [37]	(+)-7-epi-Altholactone [37]	(+)-8-epi-Altholactone [37]
H-2	4.79 (d), *J* =7.5 Hz	4.79 (d), *J* = 7.5 Hz	4.78 (d), *J* = 7.3, 4.88 Hz	5.06 (d), *J* = 4.4 Hz	5.35 (d), *J* = 2.0 Hz
H-3	4.29 (dd), *J* = 5.0, 8, 13.5 Hz	4.28 (dd), *J* = 5.5, 8 13.5 Hz	4.26 (dd), *J* = 5.4, 7.3 Hz	4.52 (t), *J* = 4.4 Hz	4.50 (d), *J* = 2.0 Hz
H-3a	5.08 (t), *J* = 5.0, 10.0 Hz	5.07 (t), *J* = 5.5, 5.5 Hz	5.04 (dd), *J* = 5.4, 5.9 Hz	5.22 (dd), *J* = 4.4, 7.8 Hz	5.08 (dd), *J* = 2.0, 4.90 Hz
H-7a	4.88 (dd), *J* = 5.0, 10.0 Hz	4.89, (t), *J* = 4.5, 5.5 Hz	4.88 (dd), *J* = 4.4, 5.9 Hz	4.78 (dd), *J* = 1.0, 3.4, 7.8 Hz	4.88 (t), *J* = 4.90, Hz
H-6	6.62 (d), *J* = 10.0 Hz	6.22 (dd), *J* = 0.7, 9.95 Hz	6.20 (d), *J* = 9.30 Hz	6.11(dd), *J* = 1.0, 10.3 Hz	6.20 (d), *J* = 9.0 Hz)
H-7	6.90 (dd), *J* = 5.0, 10.0 Hz	6.89 (dd), *J* = 4.5, 9.95 Hz	6.87 (dd), *J* = 4.4, 9.30 Hz	6.85 (dd), *J* = 3.4, 10.3 Hz	7.00 (dd), *J* = 4.90, 9.80 Hz
H-1′ to H-6′	7.34 - 7.38 (m)	7.48 (m)	7.30 - 7.38 (m)	7.20 - 7.39 (m)	7.33 - 7.43 (m)

Table S3 ^{1}H (500 MHz) and ^{13}C NMR (125 MHz) and HMBC spectroscopic data of **2** recorded in $CDCl_3$

Carbon	^{13}C	Hydrogen	^{1}H	HMBC	
				^2J	^3J

**C-2**	**83.5**	**H-2**	**4.79 (d), *J* = 7.5 Hz**	C-3	C-7a
C-3	78.8	H-3	4.29 (dd), *J* = 8 Hz, 13.5 Hz		C-7a
C-3a	78.7	H-3a	5.08 (t), *J* = 5.0 Hz, 10.0 Hz	C-3	
C-5	160.9				
C-6	141.6	H-6	6.90 (d), *J* = 10.0 Hz	C-7	C-7a
C7	123.2	H-7	6.26, *J* = 5.0 Hz, 10 .0 Hz	C-7a	
C-7a	67.8	H-7a	4.88 (t), *J* = 4.5 Hz, 10.0 Hz,	C-7	
C-1′	138.5				
C-2′, C-6′	125.9	H-2′, H-6′	7.38		
C-3′, C-5′	128.6	H-3′, H-5′	7.36 (m)		
C-4′	128.9	H-4′	7.34		

The UV spectrum supported the functional groups described above peaks with maxima at 217 (carbonyl) and 280 nm (benzene ring). The FT-IR spectrum also showed a C=O stretching band for a carbonyl group (1750 cm^{-1}). Other absorption bands included a broad band for O-H (3350 cm^{-1}), alkyl C-H stretching (2810 cm^{-1}, 2830cm^{-1} and 2942 cm^{-1}), O-H bend (890 cm^{-1}, 900 cm^{-1}1400 cm^{-1} and 1390 cm^{-1}) and C-O bend (1210 $cm^{-1,}$ 1290 - 1300 cm^{-1}).

The stereochemistry of the compound was determined by comparing the size of the coupling constant from the vicinal coupling of the hydrogens on the chiral centres with that produced by a 6-membered ring cyclohexane [34].

Based on a literature search, **2** is proposed as an unknown compound since there are only three other known stereoisomers and the compound does not completely match any of them (**Table 3**) in terms of ^{1}H and ^{13}C chemical shifts as well as the stereochemistry determined using the size of the coupling constant of the vicinal coupling between the hydrogens on the chiral centres.

Compound **3** is proposed to be 2,6-dimethoxyisonicotinaldehyde and is a new alkaloid. The compound was isolated as a colourless solid compound from the chloroform extract and stained orange with Dragendorff's reagent. The yield was 0.54% (3 mg). The HRMS gave an ion peak $[M-H]^-$ at 166.0 indicating a molecular formula $C_8H_9O_3N$ and the molecular weight of the compound was ($C_8H_9O_3N$ calcd. as 167.06).

The ^{1}H NMR spectrum (**Table S4**) showed 9 hydrogens - 2 of them were equivalent aromatic hydrogens at δ_H7.16 (H-3, H-5), another 6 hydrogens belonging to 2 equivalent methoxys (δ_H to 3.99 ppm – H_3-8 and H_3-9) while there was a methine at signal δ_H 9.82 (H-7) which was attributable to an aldehyde group (C-7). The ^{13}C NMR spectrum (**Table S4**) showed 5 carbons – 4 of them were aromatic carbons at δ_C 147.5 (C-4), 128.9 (C-3 and C-5), 106.8 (C-2 and C-6) ppm. Apart from that, there were 2 carbons at δ_C 56.7 ppm, which were part of the methoxy groups and another carbonyl carbon at δ_C 190.9 (C-7) ppm attached to the aromatic ring at C-1.

Table S4. ^{1}H (500 MHz),^{13}C NMR (125 MHz) and HMBC spectroscopic data of **3** and **4** recorded in $CDCl_3$.

**Compound 3**				
**Carbon**	**^{13}C**	**Hydrogen**	**^{1}H**	**HMBC**

				^2J	^3J	^4J
**N**						
C-4	147.5			C-3, C-5		
C-2, C-6	128.9			C-3, C-5		C-7
C-3, C-5	106.8	H-3	7.16 (s)	C-2, C-6	C-7	
C-7-CHO	190.9	H-7	9.82 (s)			
C2, C6 – OCH_3	56.7	H_3-8 , H_3-9	3.98 (s)		C-2, C-6	

Compound 4				
				HMBC
Carbon	^{13}C	Hydrogen	^{1}H	^2J or ^3J
**C-1**	**35.8**	**H_2-1**	**2.81**	C-6′
C2 – C-6		H_2-2 – H_2-5		
C-7	27.4	H_2-7	1.98	C-8
C-8	130.0	H-8 – H-9	5.29	
C-9	129.9	H-9	5.34	
C-10	27.4	H_2-10	1.98	C-9
C-11 - C-15		H_2-11– H_2-15		
C-16	32.0	H_2-16	1.47	C-17
C-17	14.3	H_3-17	0.88	C-16
C-1′	146.2			C-2′, C-6′
C-2′	115.2	H-2′	7.11 (s)	C-6
C-3′				
C-4′	122.4	H-4′	6.68 (s)	C-1
C-5′	161.2			C-6, C-4′
C-6′	133.5	H-6′	6.61 (s)	C-1
C-7′	176.1			
3′-OH				
7′-COOH				

In the HMQC spectrum, the 2 methoxy groups were attached to the aromatic ring at C-2 and C-6 whilst the HMBC spectrum further confirmed this as the 6 equivalent hydrogens from the two methoxy groups coupled with C-2 and C-6 via ^3J correlations. Additionally, C-7 (the aldehyde moiety) was coupled to H-3 and H-5 in the aromatic ring via ^3J correlation. C-3 coupled to H-5 and H-7 and C-5 coupled with H-3 and H-7, both via ^3J correlation as shown in the HMBC spectrum. The HMBC spectrum further indicated that there were ^2J couplings between the tertiary carbon (C-4) with H-3 and H-5.

The FT-IR spectrum was taken by dissolving it in methanol and showed a C=N double bond (1650cm^{-1}) and aldehyde stretching (1730cm^{-1}). There was an alkyl C-H stretching (2815 cm^{-1} and 2940 cm^{-1}) as well as C-O bend (1205 $cm^{-1,}$ 1297 cm^{-1}).

The UV spectrum showed conjugation with peaks at maxima 220 (237.7) and 238 (70.4) (aldehyde) nm.

Based on the above data, **3** is proposed as a new alkaloid, **2,6-dimethoxyisonicotinaldehyde**.

Compound **4** is proposed as (*Z*)-3-(heptadec-8-en-1-yl)-5-hydroxybenzoic acid which is colourless and was isolated from an ammonia extract using dichloromethane by means of back extraction and the yield was 15 mg (20.5% of the fraction). The double bond of the compound had a small coupling constant (<4 Hz) and therefore is *cis*. The HRMS gave an ion peak $[M-H]^-$ at *m/z* 373.2732 which corresponded to the molecular formula $C_{24}H_{38}O_3$ (calculated as 374.2822).

The ^1H NMR spectrum (**Table S4**) showed 38 hydrogens including 14 pairs of methylenes, a methyl triplet at δ_H 0.88 ppm as well as 2 olefinic hydrogens at δ_H 5.29 (H-8) and 5.34 (H-9) ppm of the long alkyl chain, three singlet aromatic hydrogens at δ_H 6.61, 6.68 and 7.11 ppm and 2 hydrogens which were part of the hydroxyl and carboxylic groups attached to the aromatic ring while the ^{13}C NMR spectrum (**Table S4**) showed 24 carbons including 14 methylene carbons (C-1 – C7, C-10 – C-16), 2 olefinic carbons at δ_C 129.9 and 130.0 ppm and a methyl carbon (C-17) at δ_C 14.3 ppm attributable to part of the long alkyl chains as well as 3 aromatic methane carbons signals at δ_C 133.5 (C-2′); 122.3 (C-4′) and 115.2 (C-6′) ppm, a deshielded aromatic quaternary carbon (C-5′) bearing an hydroxyl (δ_C 161.2 ppm), a further aromatic quaternary carbon (C-3′, δ_C 146.5 ppm) bearing a carboxylic group (C7′, δ_C 176.1 ppm).

The position of the double bond cannot be ascertained using NMR spectra and was therefore determined using GC/MS as employed by Shibamoto [5]. A fragment from the EI mass spectrum obtained from the GC/MS matched the alkenyl chain of the compound **4** which showed that the double bond is positioned between C-8 and C-9 (**Figure S31**).

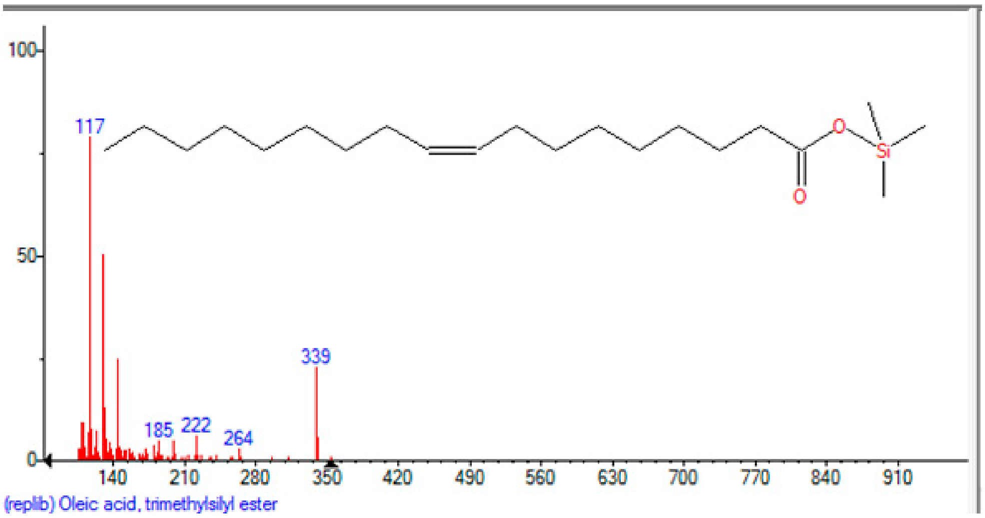

Figure S31. The EI mass spectrum of the GC peak corresponding to the alkenyl chain of **4** as identified from the NIST database (fragmentation pattern proposed as oleic acid trimethylsilyl acid).

The structure was further confirmed by the coupling of each hydrogen to the 2nd or 3rd neighbouring carbons. In the HMBC spectrum, the alkenyl chain was coupled to the aromatic ring via two bonds. H-1 showed ^3J correlations with C-6′/C-2′ of the aromatic ring whilst the carboxylic moiety was attached to the aromatic ring at C-3′ which was supported by correlations between H-2′ and H-4′ to C-7′. Furthermore, H-2′

and H-6′ also exhibited ^{2}J correlation with C-2′ and C-6′ whilst C-5′ coupled via ^{2}J correlation with C-4′ and C-6′.

Further confirmation of the structure of the compound was obtained using UV–visible and FT-IR spectroscopic techniques, which showed the presence of a C=C double bond at peak 217 nm and a benzene ring at 290 nm and a C=O stretching for the carboxylic acid (1750 cm^{-1}) was observed. Further absorptions in IR spectrum was attributable to O-H (3350 cm^{-1}), alkyl C-H stretching (2810 cm^{-1}, 283 cm^{-1} and 2942 cm^{-1}), O-H bend (890 cm^{-1}, 900 cm^{-1}, 1400 cm^{-1} and 1390 cm^{-1}) and C-O bend (1210 cm^{-1}, 1290 cm^{-1} and 1300 cm^{-1}) were also present.

Based on the above data, **4** is proposed as the new natural product (Z)-3-(heptadec-8-en-1-yl)-5-hydroxybenzoic acid.

1.1. Determination of the location of double bonds in alkenyl chains and the derivatization protocol

The Gas chromatography-mass spectrometry (GCMS) together with EI ionization method and helium gas as the mobile phase. In order to determine the location of a double bond in a long chain such as in alkenyl phenols, there is a need to prepare a dimethyl disulfide (DMDS) derivatives (adducts) from the compound. If a hydroxyl group is present, then there is a need to perform derivatization first as the hydroxylated compound group has poor chromatographic properties in Gas Chromatography (GC).

The preparation of the dimethyl disulphide derivative is shown in **Figure S32**.

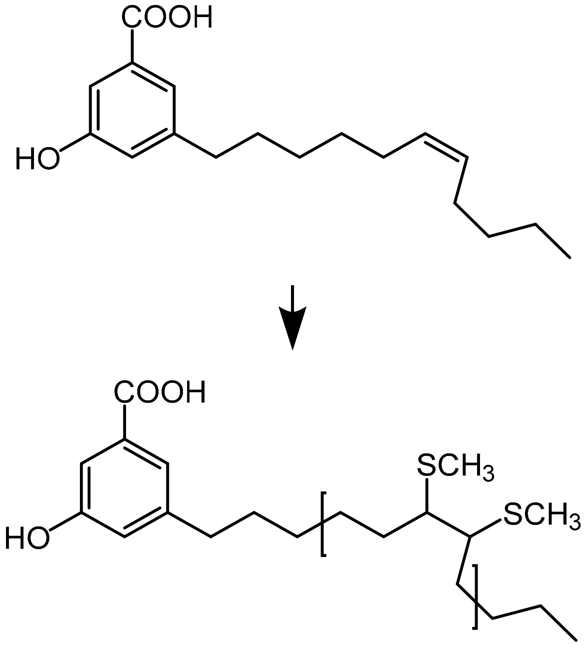

Figure S32. Preparation of dimethyl disulphide derivatives

To the DMDS adducts, 200 μL of bis(trimethylsilyl)trifluoroacetamide and trimethylchlorosilane (BSTFS-TMCS 99:1) were added. The mixture was left to stand at room temperature for 30 minutes. It was then analysed using GC-MS. The derivatisation of the hydroxyl group is shown in **Figure S33**

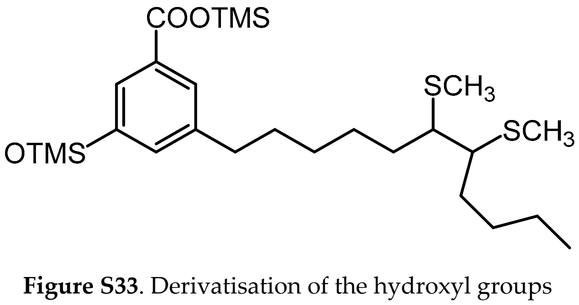

Figure S33. Derivatisation of the hydroxyl groups

References

1. Loder, J.W.; Nearn, R.H. Altholactone: a novel tetrahyfrofur[3,2b] pyran-5-one from a *Polyathia* species. *Heterocycl*. **1997**, *7*, 113-118.
2. Colegate, S.M.; Laily, B.D.; Latiff, A.; Mat Salleh, K.;Samsudin, M.W. ; Skelton, B.W.; Tadan, K.I.; White, A.H.;Zuriati Z.(+)-Isoaltholactone: A furanopyrone isolated from *Goniothalamus* species *Phytochem*. **1990,**, 1701-1704.doi:/10.1016/0031-9422(90)80153-8
3. Reich, H.J. 5-HMR-5 Vicinal proton-proton coupling ^{2}J H-H. 2008, available online: https://organicchemistrydata.org/hansreich/resources/nmr/#05-hmr-05-3j/ (accessed on 1 June 2020).
4. Ueno,Y., Tadano, K.-I., Ogawa, S. Total synthesis of (+)-altholactone [(+0-Goniothalenol] and three stereocongeners and their cytotoxicity against several tumor cells. *Bull. Chem. Soc. Jpn.***1989**, 62, 2328-2337.
5. Shibamoto,S.; Murata, T.; Yamamoto, K. Determination of double bond positions and geometry of methyl lineolate isomers with dimethyl disulfide adducts by GC/MS. *Lipids*. **2016**, 51, 1077-1081.
